# Editorial Note: The multi-targeted kinase inhibitor sunitinib induces apoptosis in colon cancer cells via PUMA

**DOI:** 10.1371/journal.pone.0339805

**Published:** 2026-01-06

**Authors:** The PLOS One Editors

After publication of this article [1], concerns were raised regarding Figs 1, 2, 6 and S1, specifically, the following panels appear similar to each other:

- The β-actin panels in Fig 1D and Fig S1A
- The β-actin panels in Fig 2D and Fig S1B
- The Mcl-1 panels in Fig 6B

The corresponding author stated that the β-actin panels in Fig 1D and Fig S1A, and in Fig 2D and Fig S1B, were intentionally repeated as the data are from the same experiments, where the full set of blots for each experiment with the corresponding actin blot were split between the main article [1] and Supporting Information. The underlying blots for Figs 1D, 2D, S1A and S1B are provided here in S6 and S8 Files. The corresponding author also stated that the Mcl-1 panels in Fig 6B were duplicated in error but that they do not know if either of these panels are correct given that the underlying data for Fig 6B are no longer available. They provided repeat data for Fig 6B from later experiments in S3–S4 Files, which were reviewed by a member of the *PLOS One* Editorial Board.

The corresponding author also stated that for Figure 1C, the ΔΔCt (ddCt) method for fold calculation in qPCR was used, Relative *PUMA* refers to -ΔΔCt, and “2 de mi” refers to fold conversion as 2^-ΔΔCt^.

The corresponding author provided the available underlying data supporting other published results (S1–S3 and S5–S8 Files).

## Supporting information

S1 File The original individual-level underlying data in support of Figure 5A. (XLSX)

S2 File The original individual-level underlying data in support of Figure 6D, including raw counts and conversion to percentages. (XLSX)

S3 File Original underlying blots in support of Figs 1C, 3A and 4A, and underlying blots in support of the later repeat experiments for the updated PUMA, Mcl-1 and β-actin panels in the updated Fig 6B in S4 File. (PPTX)

S4 File Updated Figure 6B with updated panels for PUMA, Mcl-1 and β-actin from later repeat experiments. (PPTX)

S5 File The original individual-level underlying data in support of Figs 1C, 2A and D, 4C-D, and 5B-C, including raw counts and conversion to percentages for Fig 2A. (XLSX)

S6 File Original and representative underlying blots in support of Figs 1B (HCT 116 β-actin and p21 panels and HCT 116 p53 KO PUMA and β-actin panels), 1D, S1A, 2D, 5B, 5C (Cas-3 panel) and 6C (p-AKT panels). (PDF)

S7 File Original underlying blots in support of Fig 4A IP panel, Fig 3D FoxO3a panel and Fig S1B Bid panel. (PPTX)

S8 File Original underlying blots in support of Fig S1B. (PDF)
