# Supplementary material for: Editorial Note: The multi-targeted kinase inhibitor sunitinib induces apoptosis in colon cancer cells via PUMA
Source: PLoS One. 2026 Jan 6;21(1):e0339805. doi: 10.1371/journal.pone.0339805 (PMC12773795; doi:10.1371/journal.pone.0339805)
Supplement: S3 File — (PPTX) [file pone.0339805.s003.pptx]

## Slide 1
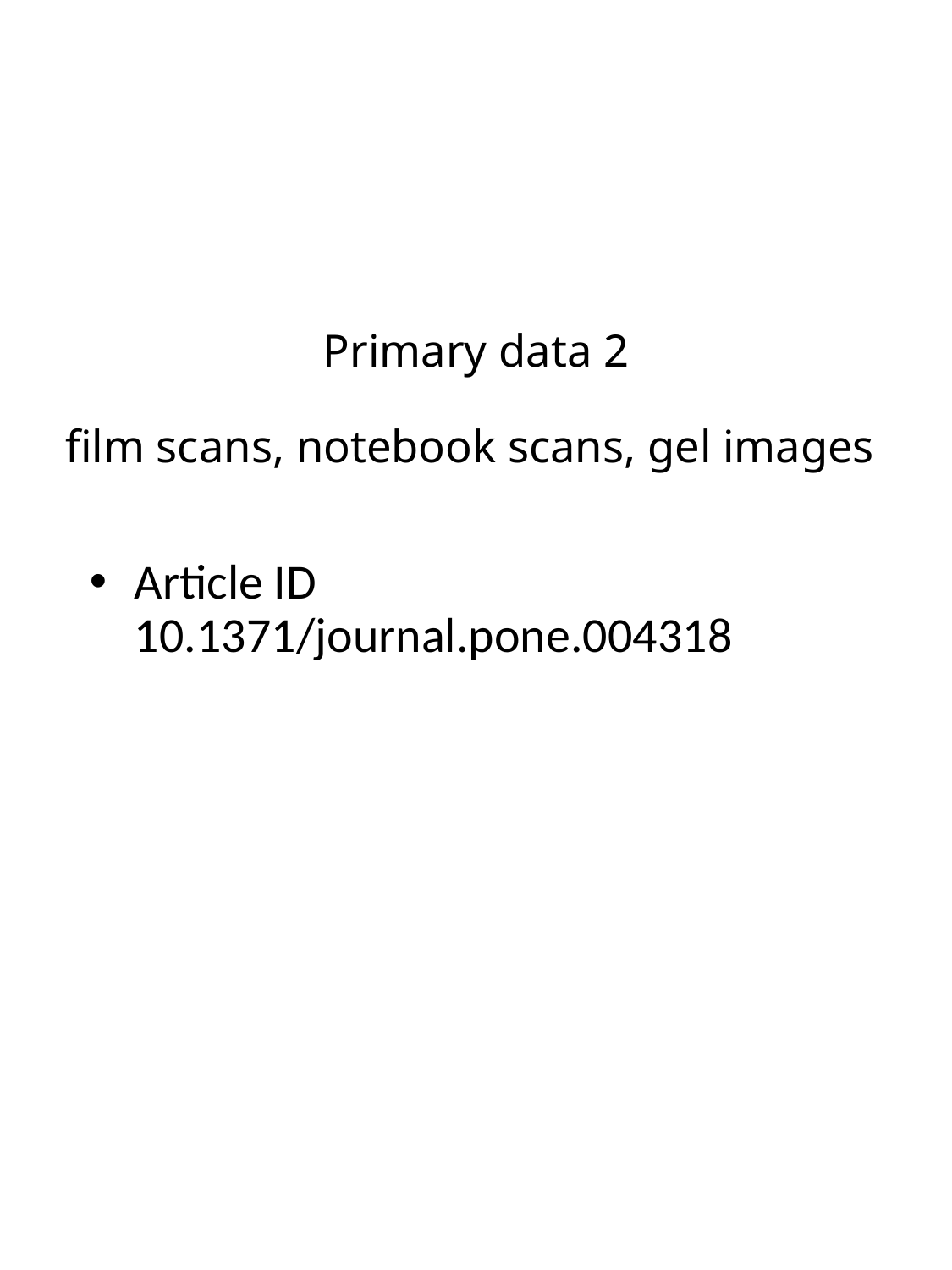

# Primary data 2 film scans, notebook scans, gel images
Article ID 10.1371/journal.pone.004318

## Slide 2
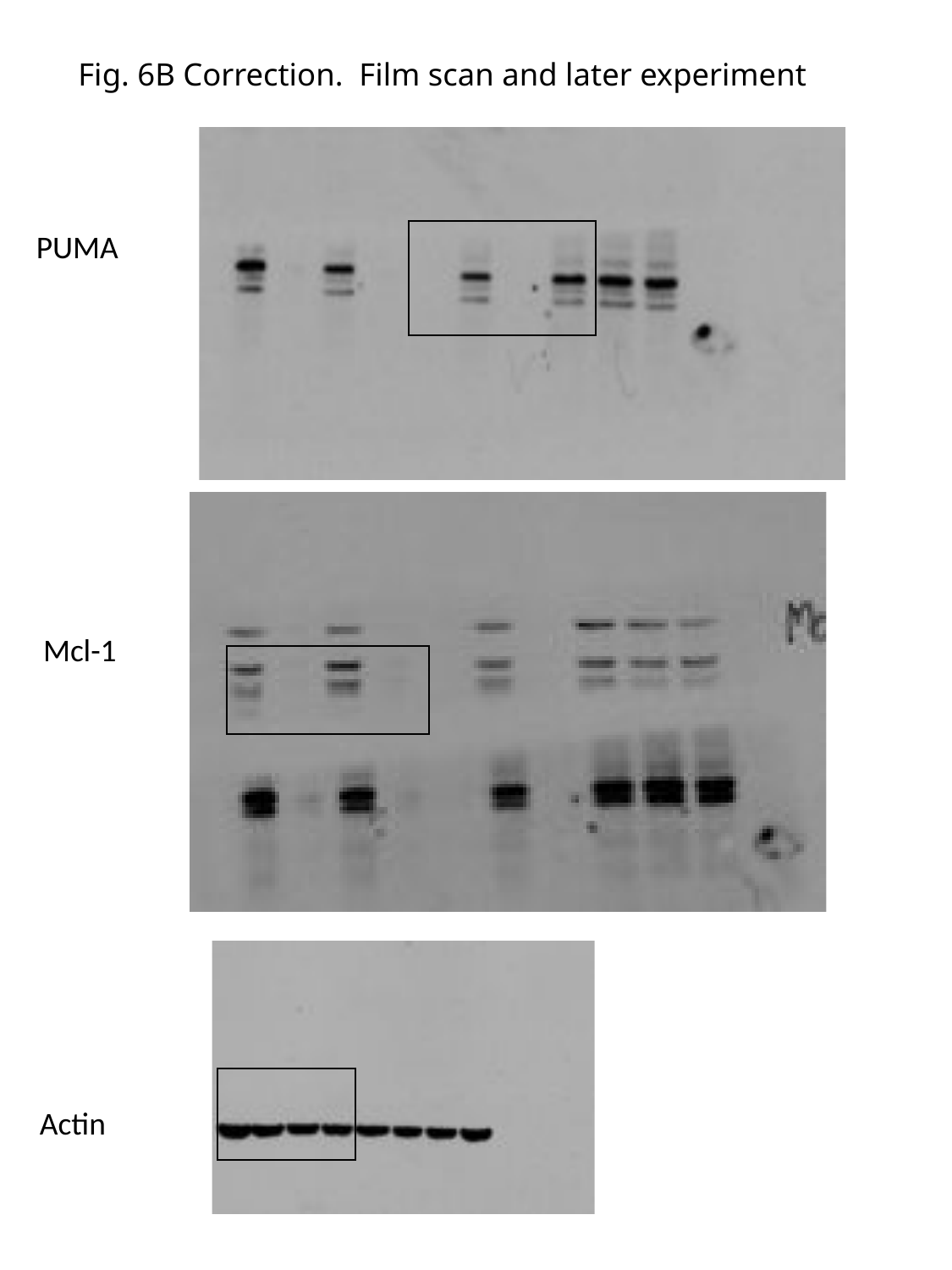

# Fig. 6B Correction. Film scan and later experiment
PUMA
Mcl-1
Actin

## Slide 3
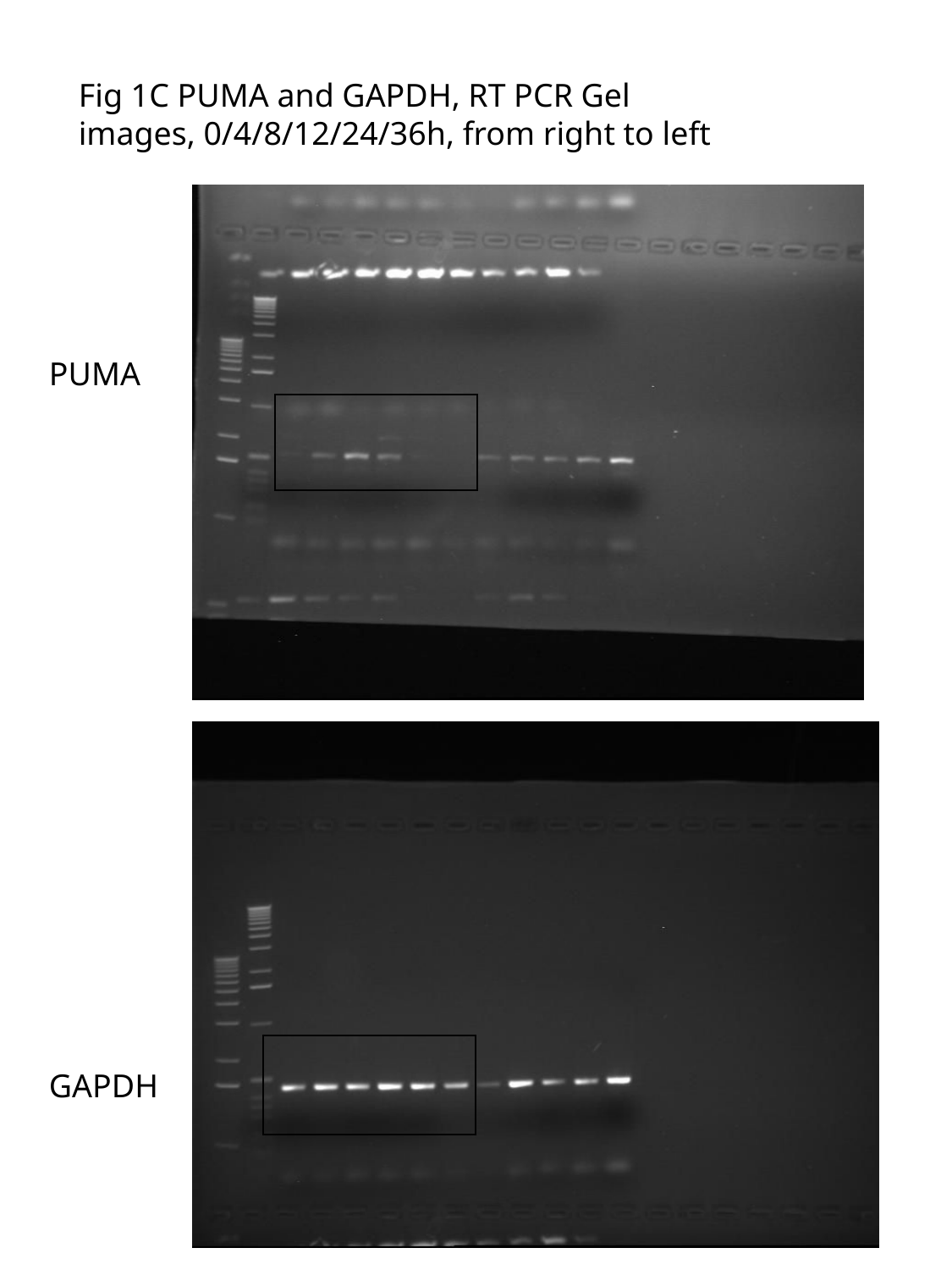

Fig 1C PUMA and GAPDH, RT PCR Gel images, 0/4/8/12/24/36h, from right to left
PUMA
GAPDH

## Slide 4
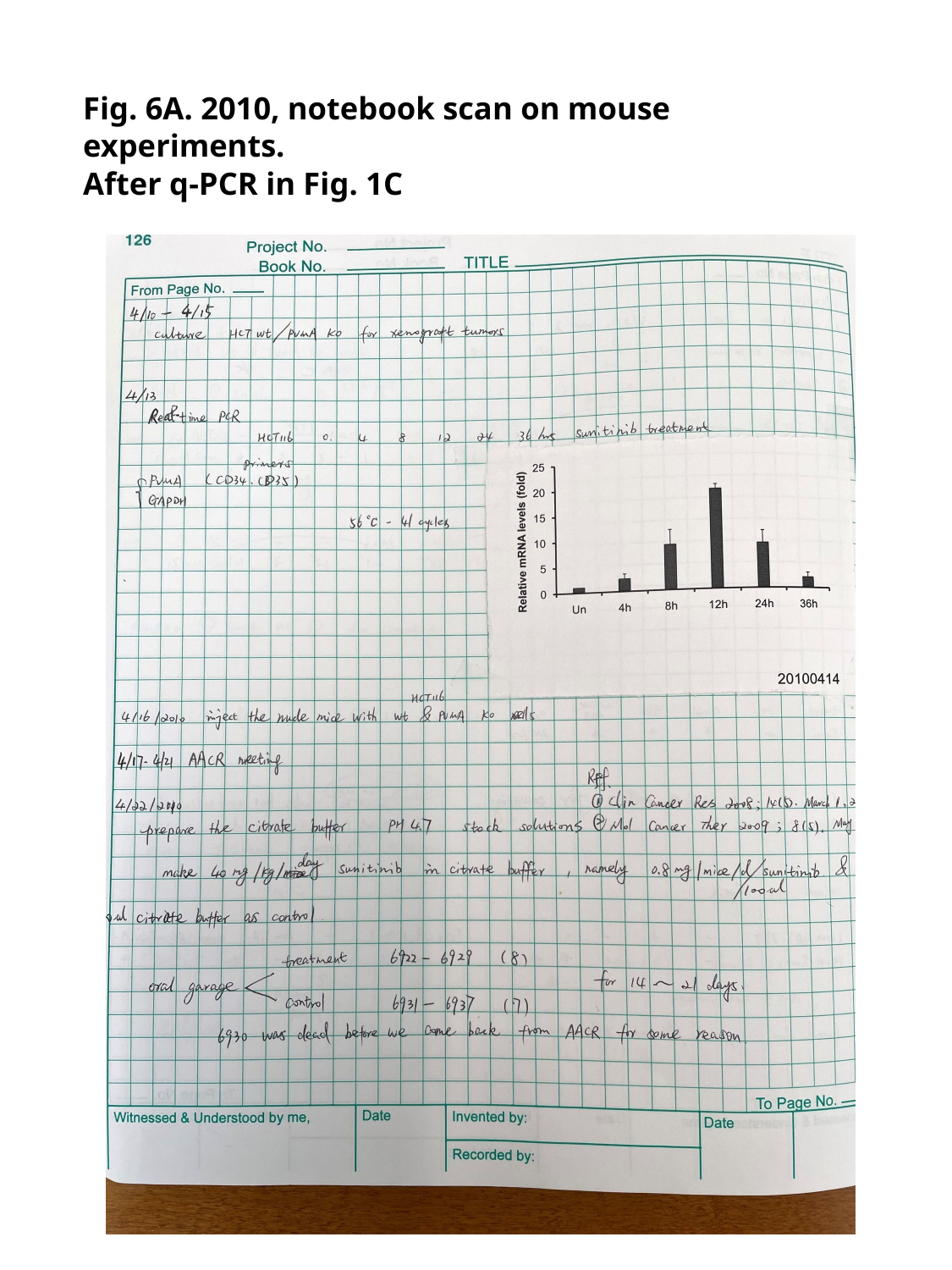

# Fig. 6A. 2010, notebook scan on mouse experiments. After q-PCR in Fig. 1C

## Slide 5
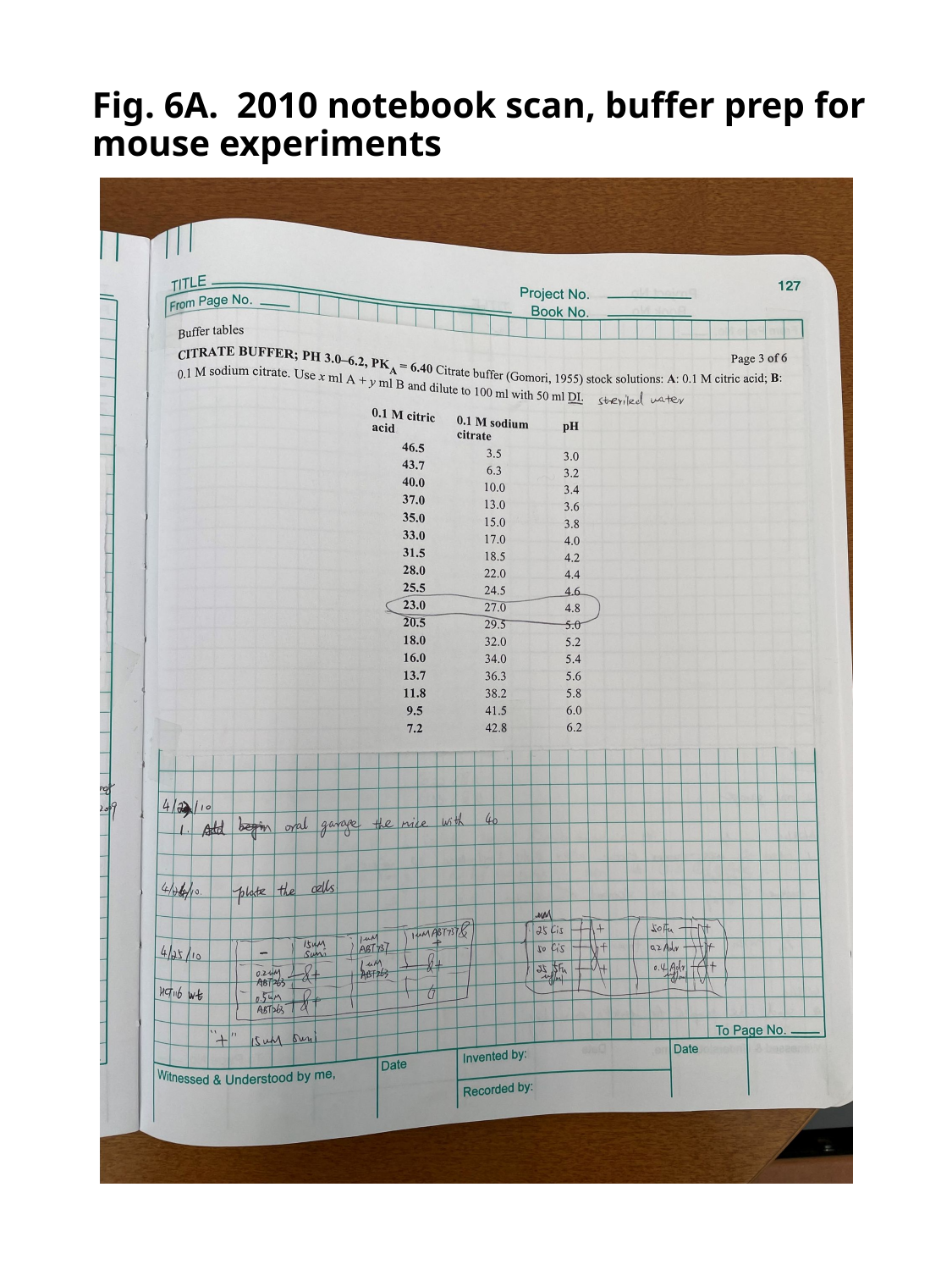

# Fig. 6A. 2010 notebook scan, buffer prep for mouse experiments

## Slide 6
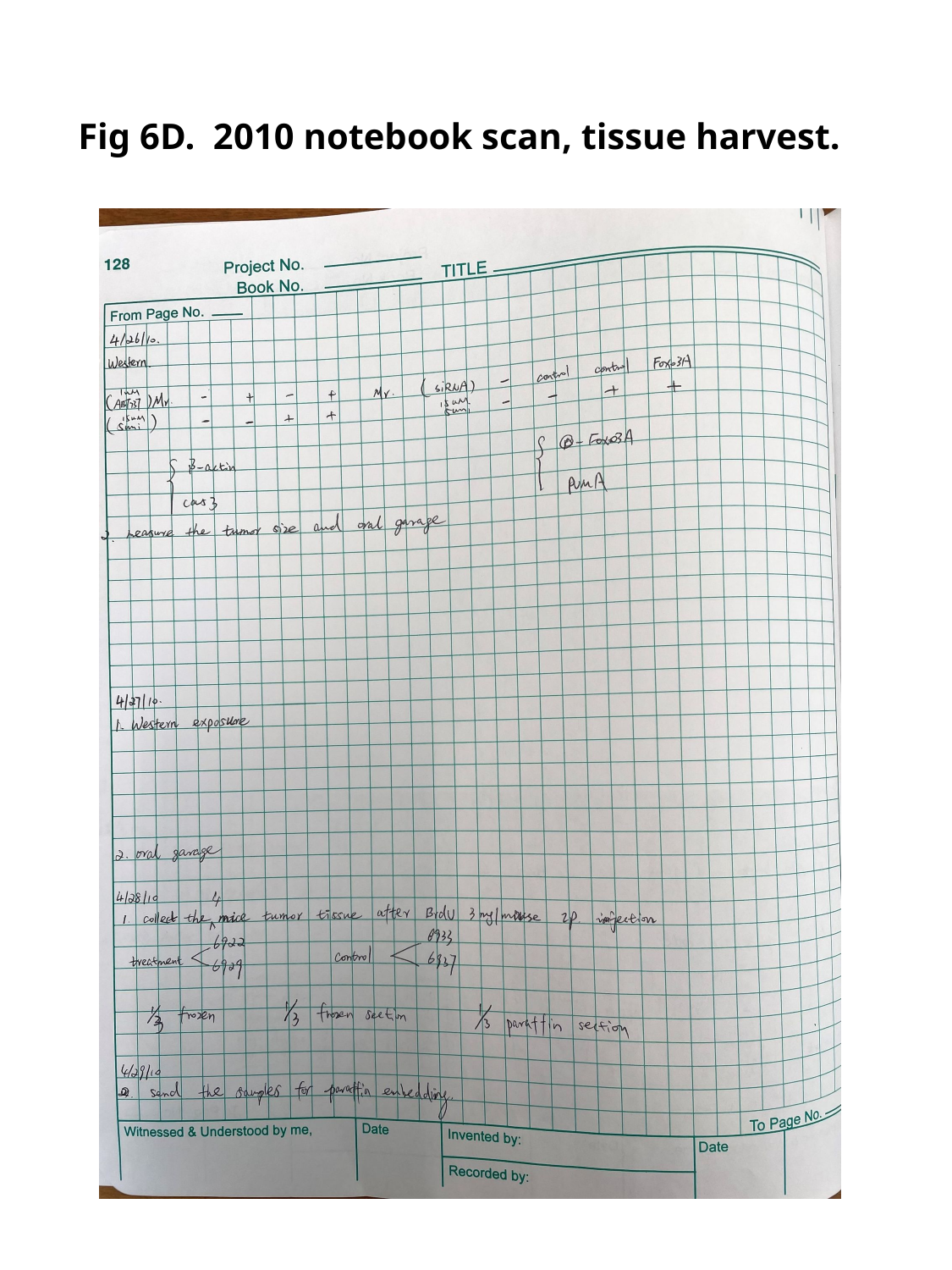

# Fig 6D. 2010 notebook scan, tissue harvest.

## Slide 7
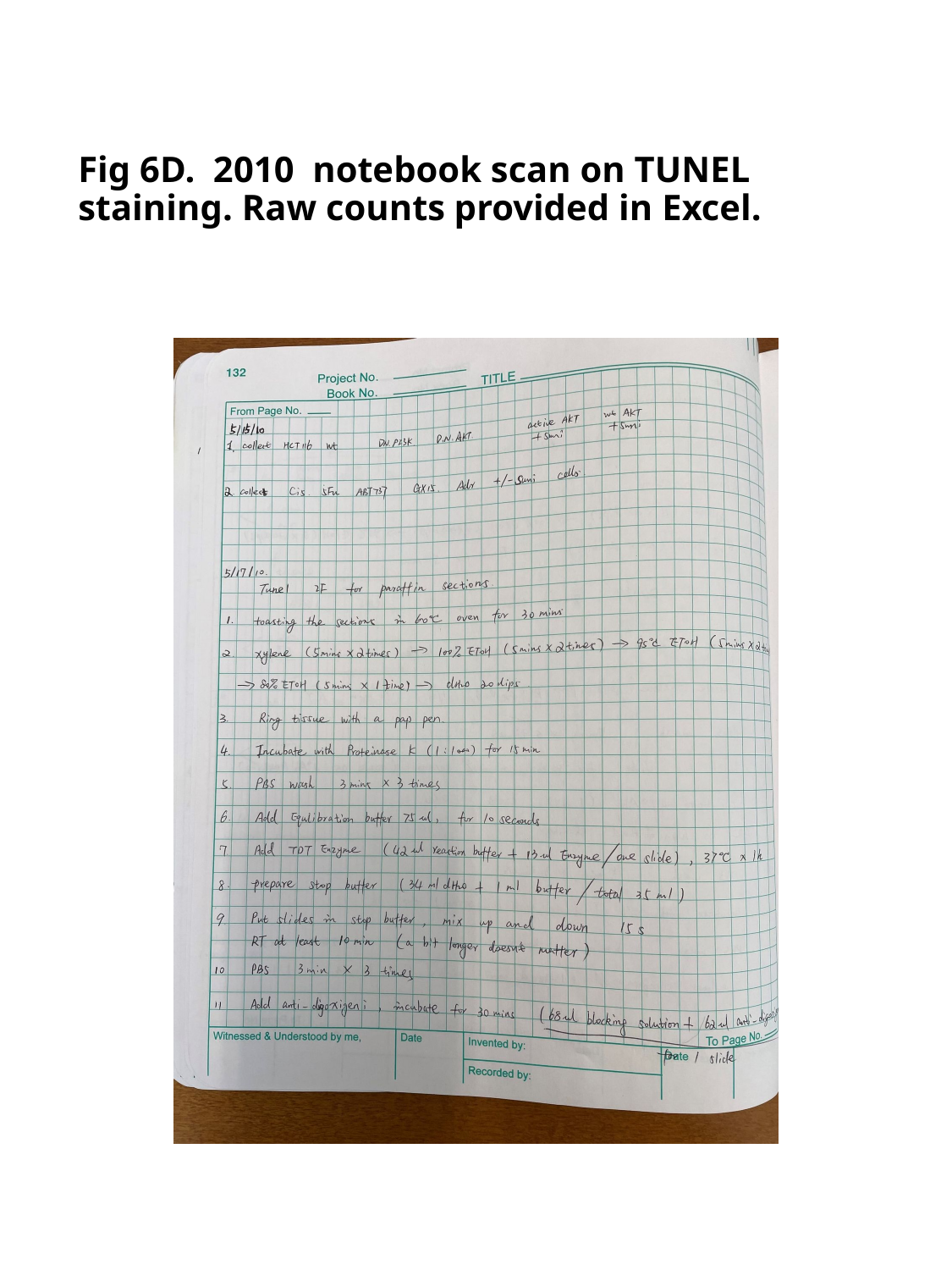

# Fig 6D. 2010 notebook scan on TUNEL staining. Raw counts provided in Excel.

## Slide 8
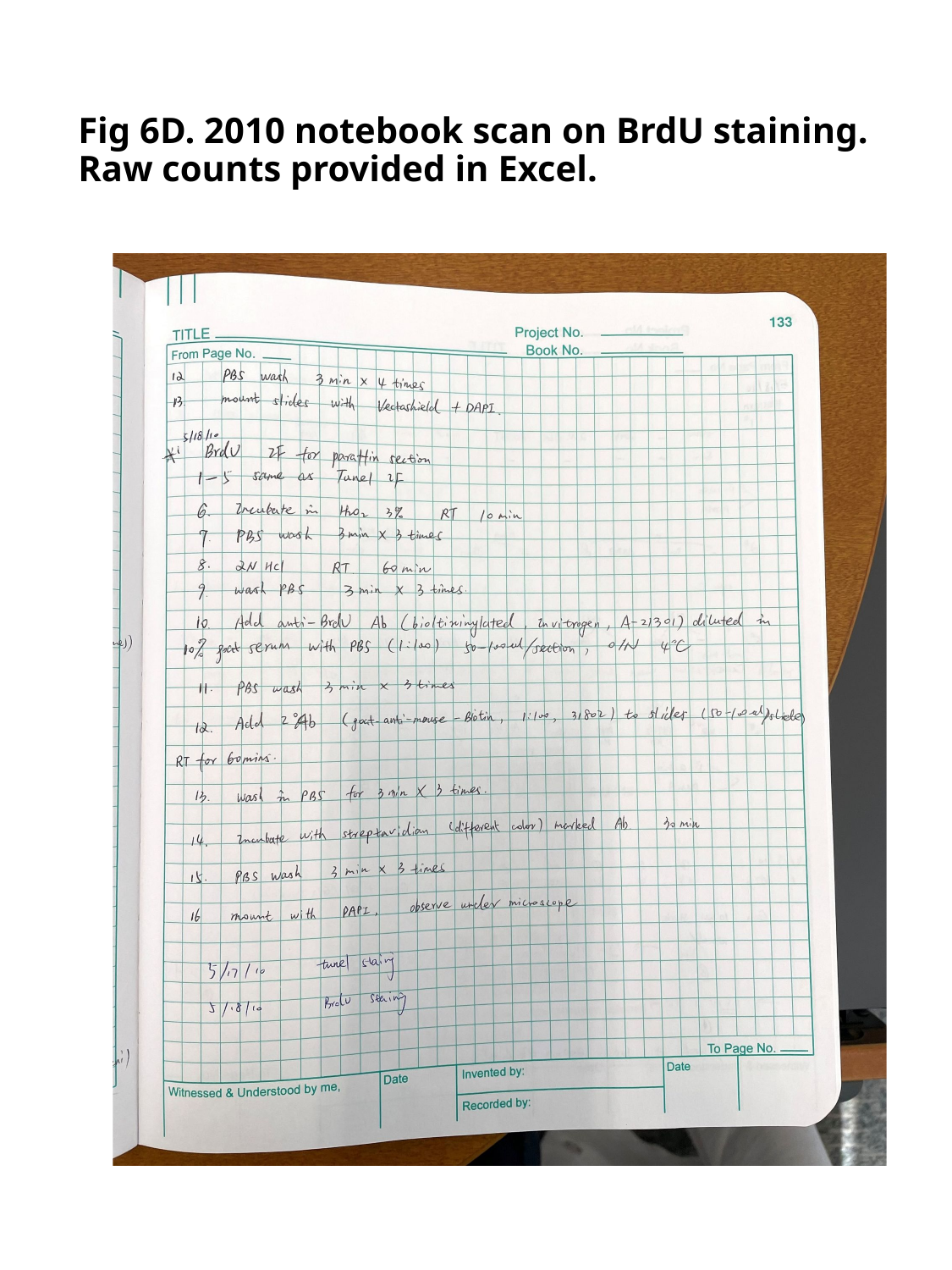

# Fig 6D. 2010 notebook scan on BrdU staining. Raw counts provided in Excel.

## Slide 9
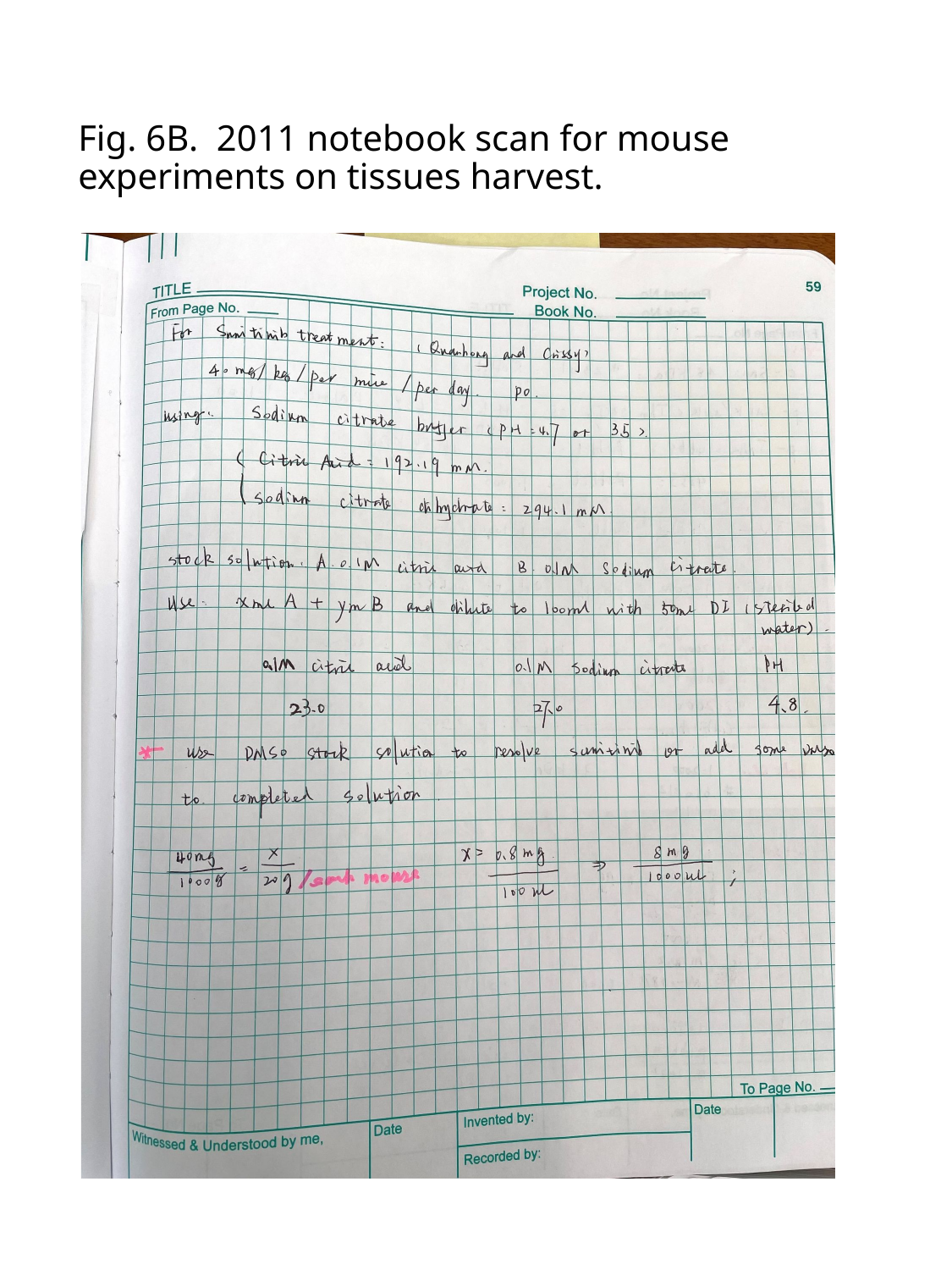

# Fig. 6B. 2011 notebook scan for mouse experiments on tissues harvest.

## Slide 10
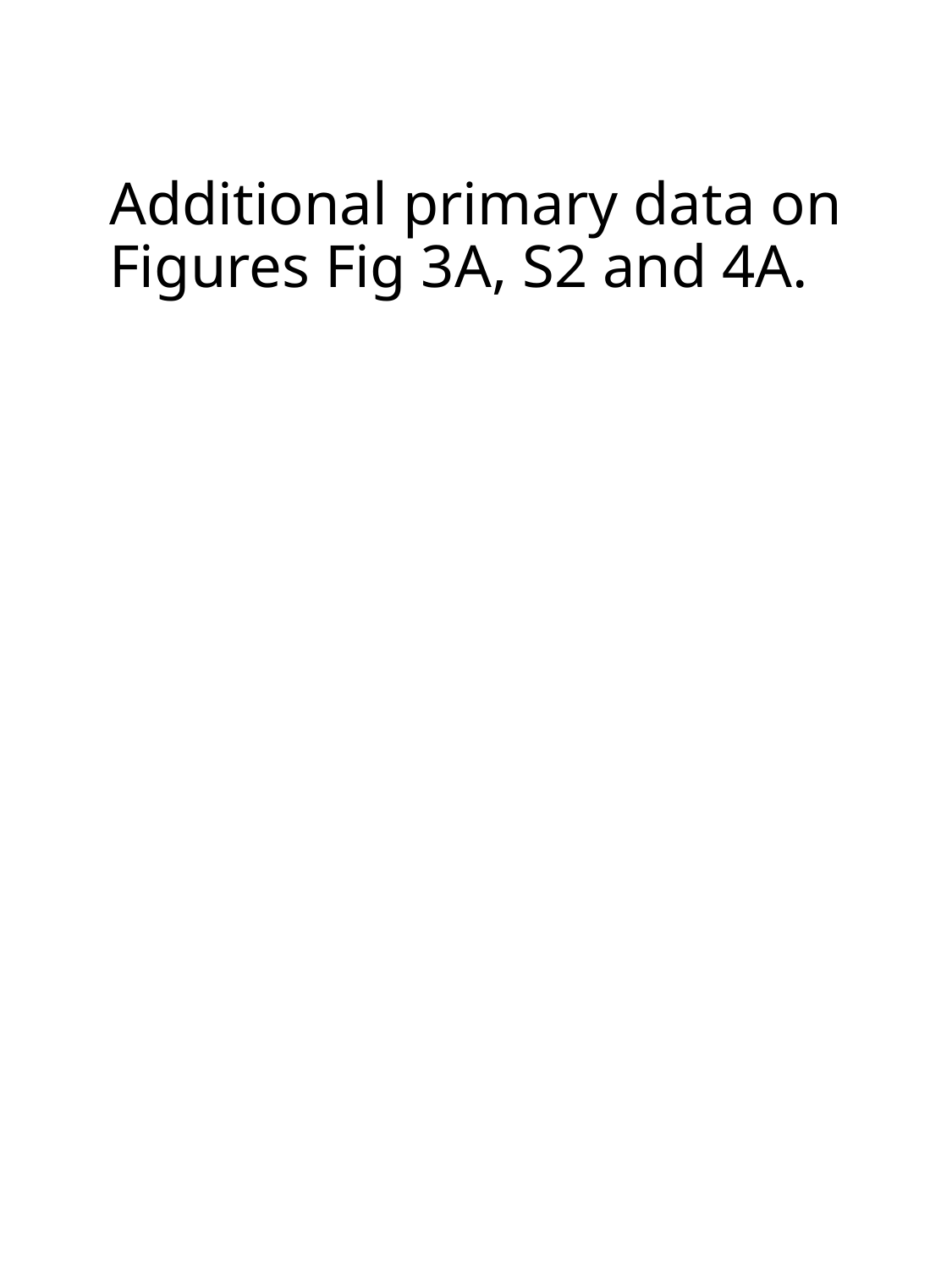

# Additional primary data on Figures Fig 3A, S2 and 4A.

## Slide 11
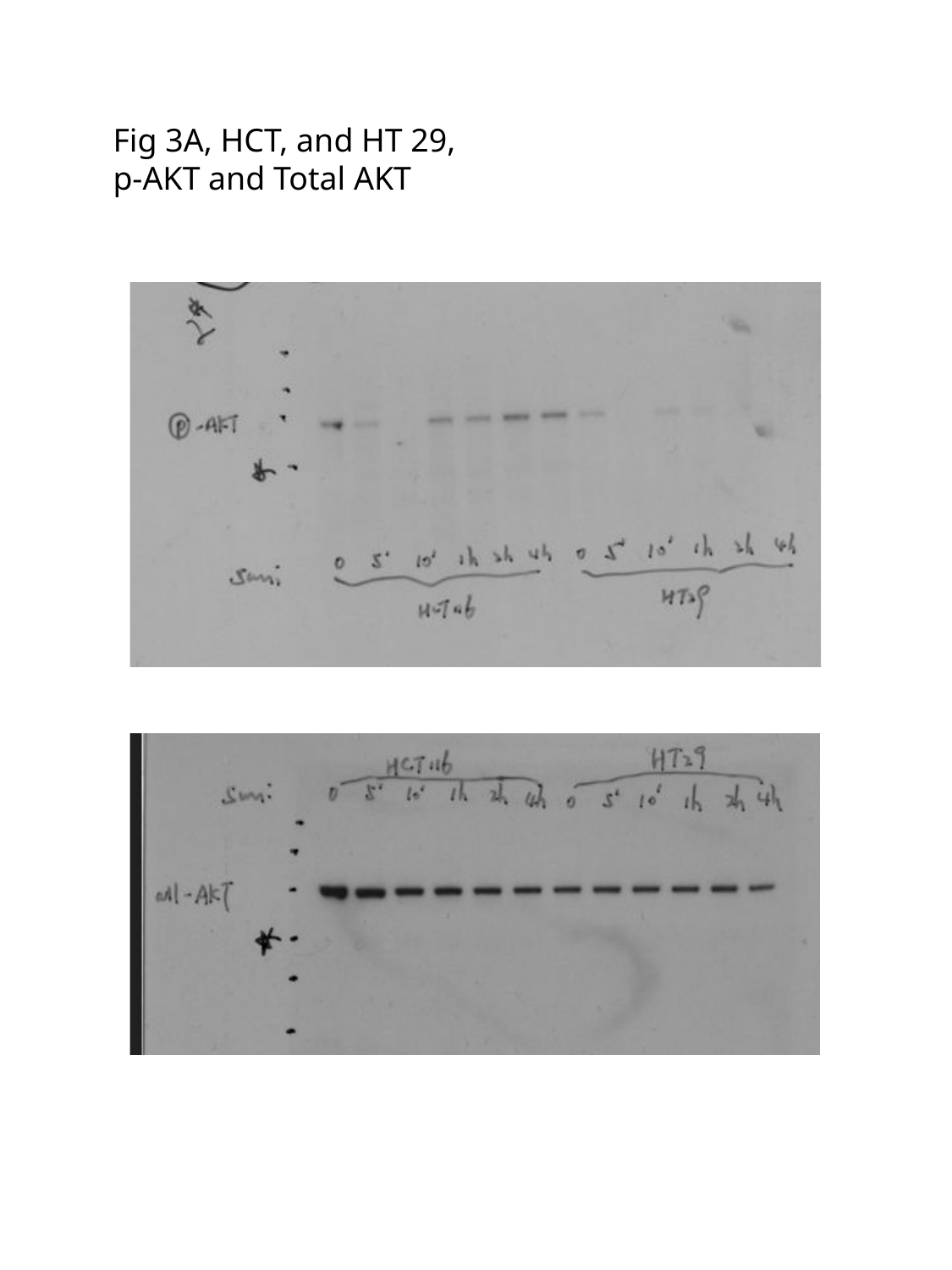

Fig 3A, HCT, and HT 29,
p-AKT and Total AKT

## Slide 12
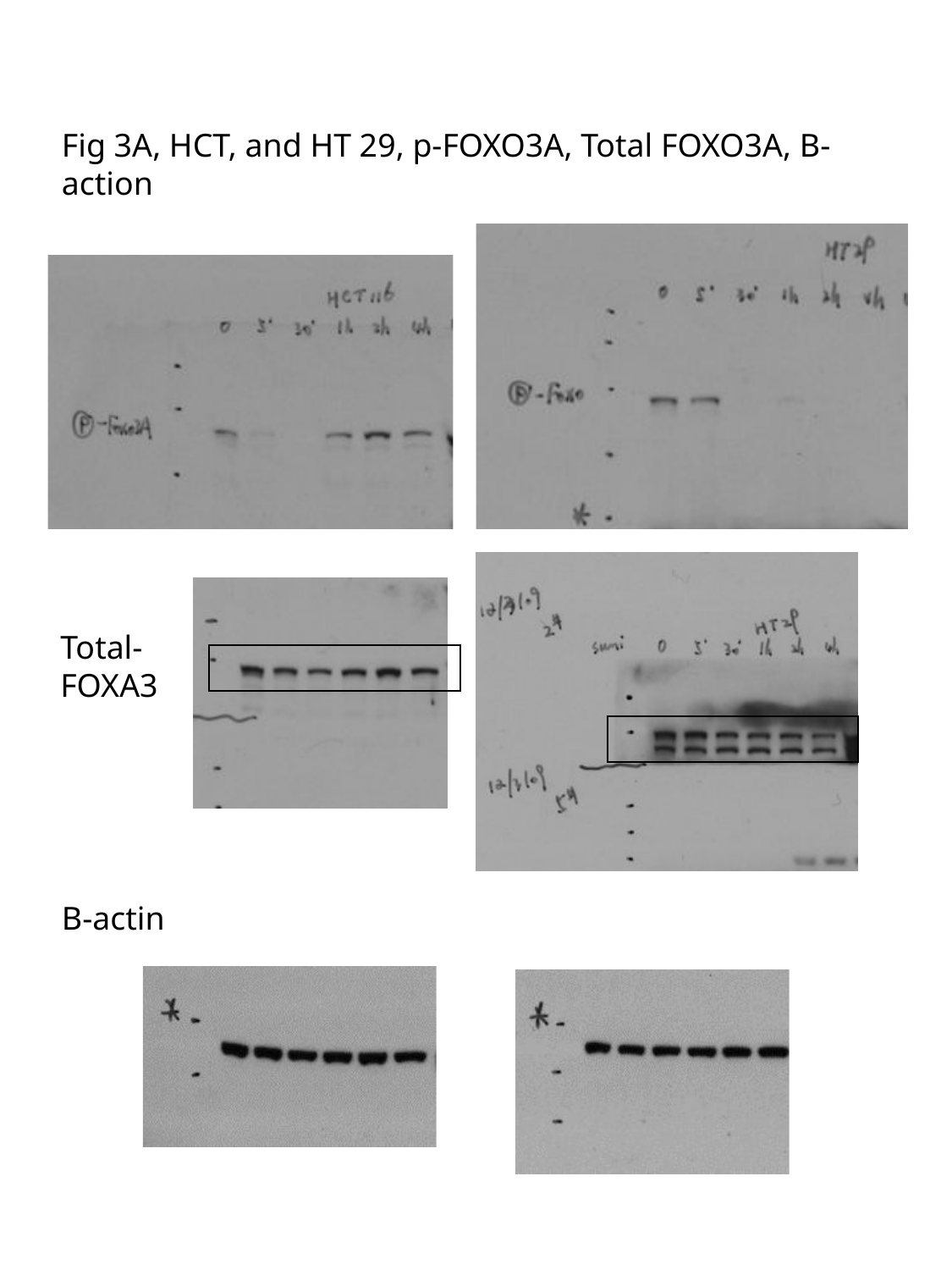

Fig 3A, HCT, and HT 29, p-FOXO3A, Total FOXO3A, B-action
Total- FOXA3
B-actin

## Slide 13
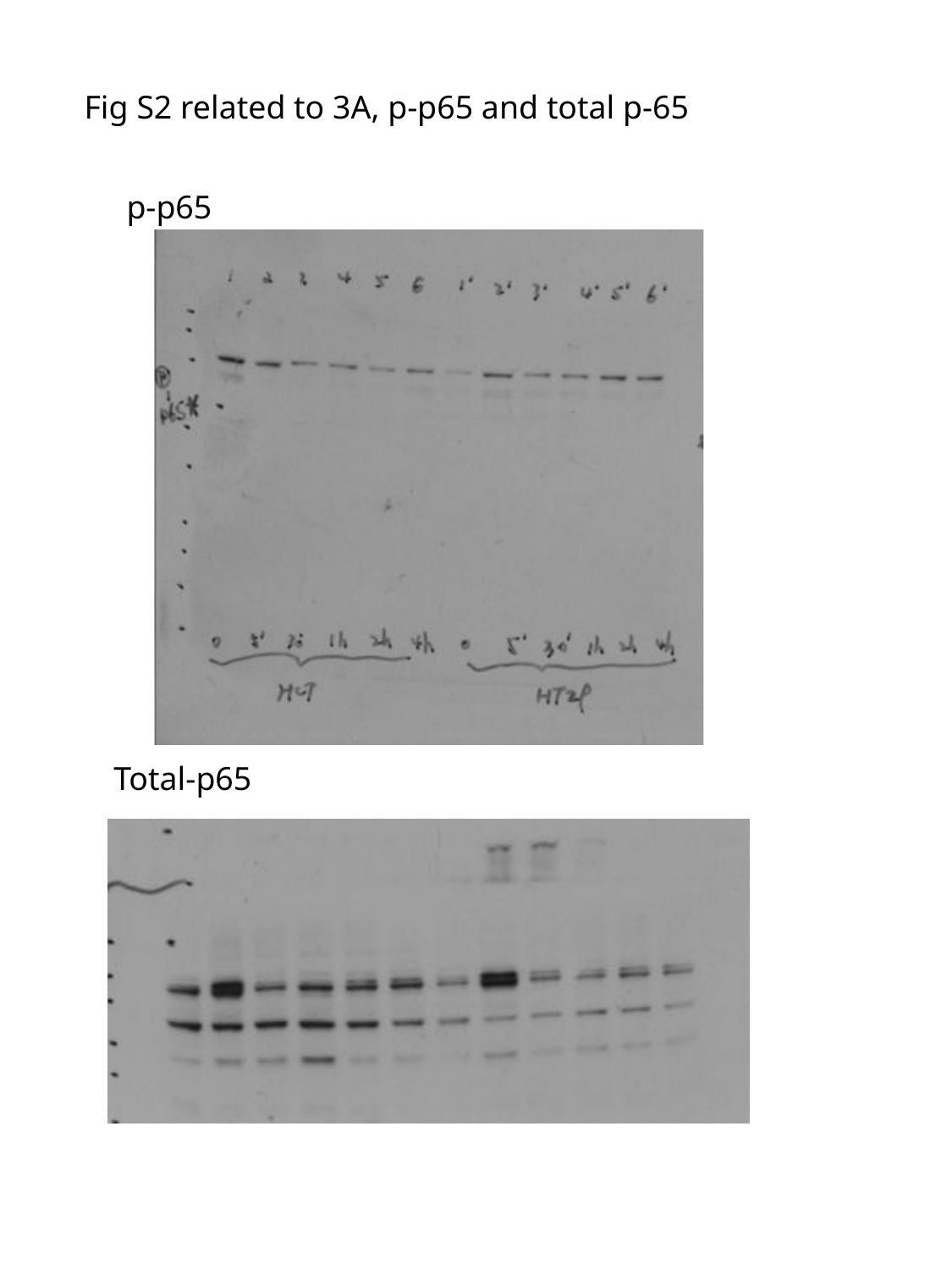

Fig S2 related to 3A, p-p65 and total p-65
p-p65
Total-p65

## Slide 14
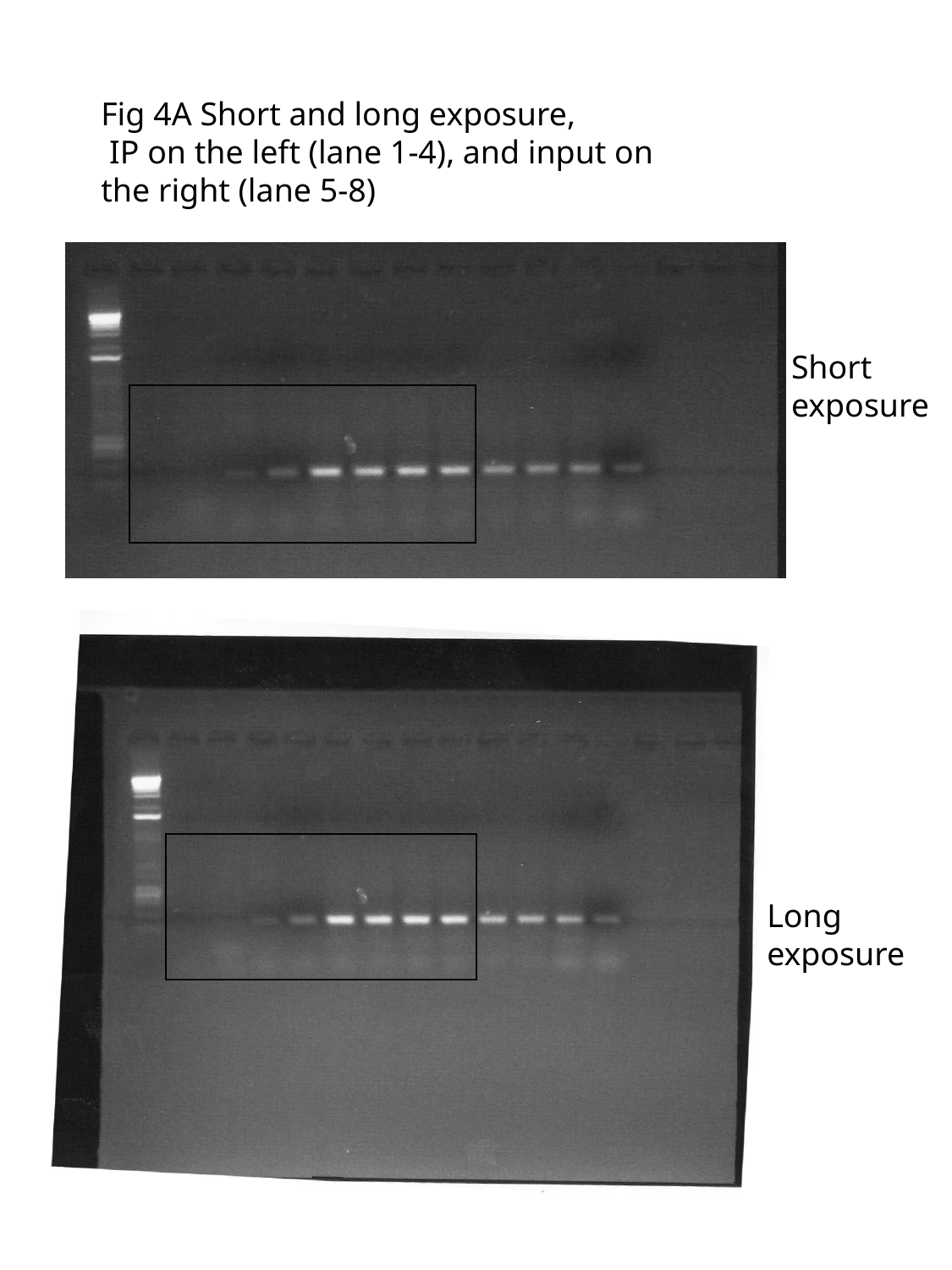

Fig 4A Short and long exposure,
 IP on the left (lane 1-4), and input on the right (lane 5-8)
Short exposure
Long exposure
