# Supplementary material for: Editorial Note: The multi-targeted kinase inhibitor sunitinib induces apoptosis in colon cancer cells via PUMA
Source: PLoS One. 2026 Jan 6;21(1):e0339805. doi: 10.1371/journal.pone.0339805 (PMC12773795; doi:10.1371/journal.pone.0339805)
Supplement: S4 File — (PPTX) [file pone.0339805.s004.pptx]

## Slide 1
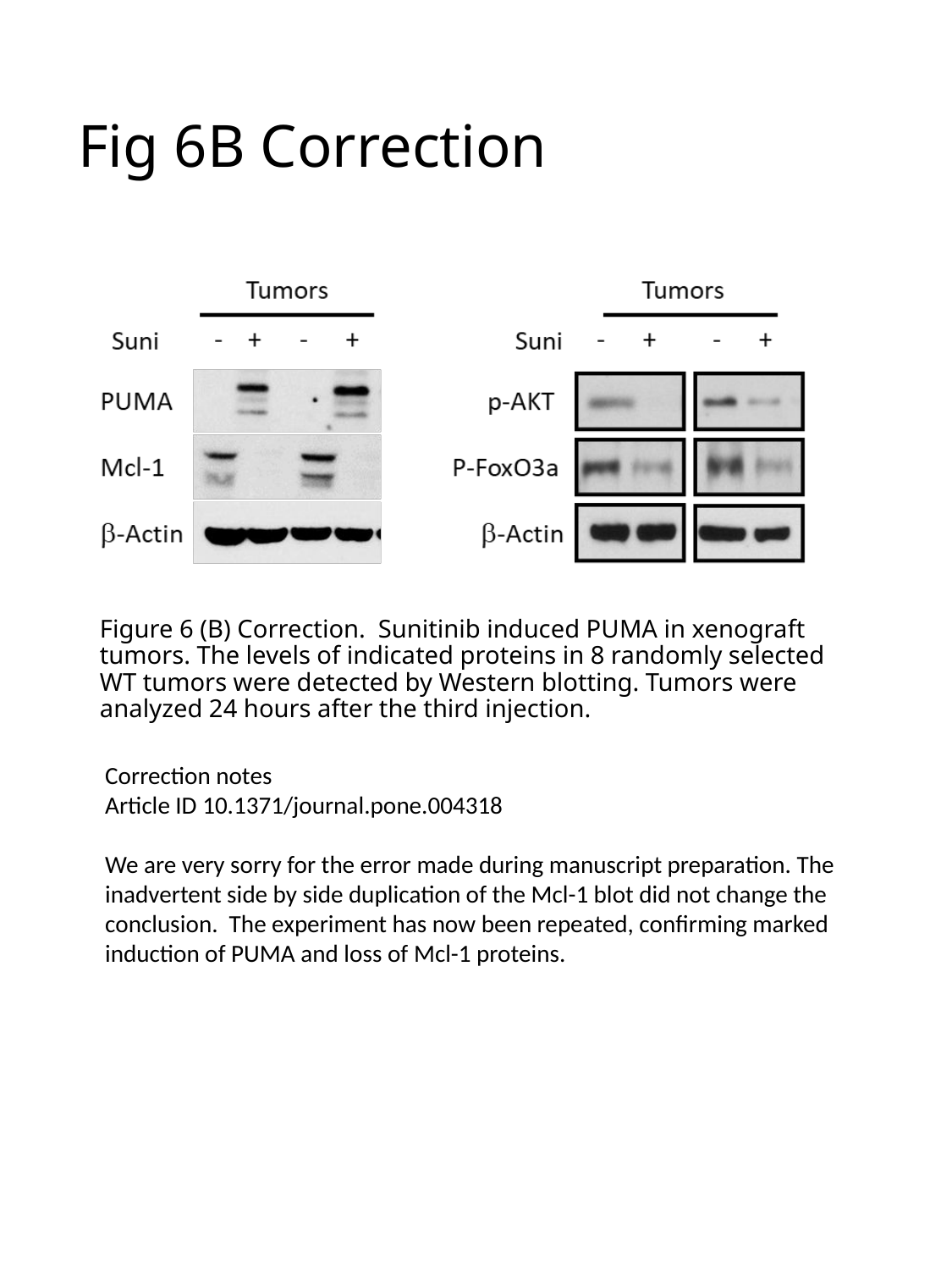

# Fig 6B Correction
Figure 6 (B) Correction. Sunitinib induced PUMA in xenograft tumors. The levels of indicated proteins in 8 randomly selected WT tumors were detected by Western blotting. Tumors were analyzed 24 hours after the third injection.
Correction notes
Article ID 10.1371/journal.pone.004318
We are very sorry for the error made during manuscript preparation. The inadvertent side by side duplication of the Mcl-1 blot did not change the conclusion. The experiment has now been repeated, confirming marked induction of PUMA and loss of Mcl-1 proteins.
