# Supplementary material for: Editorial Note: The multi-targeted kinase inhibitor sunitinib induces apoptosis in colon cancer cells via PUMA
Source: PLoS One. 2026 Jan 6;21(1):e0339805. doi: 10.1371/journal.pone.0339805 (PMC12773795; doi:10.1371/journal.pone.0339805)
Supplement: S6 File — (PDF) [file pone.0339805.s006.pdf]

Scans film/notebook and images

Article ID 10.1371/journal.pone.004318

Fig. 1B notebook scan

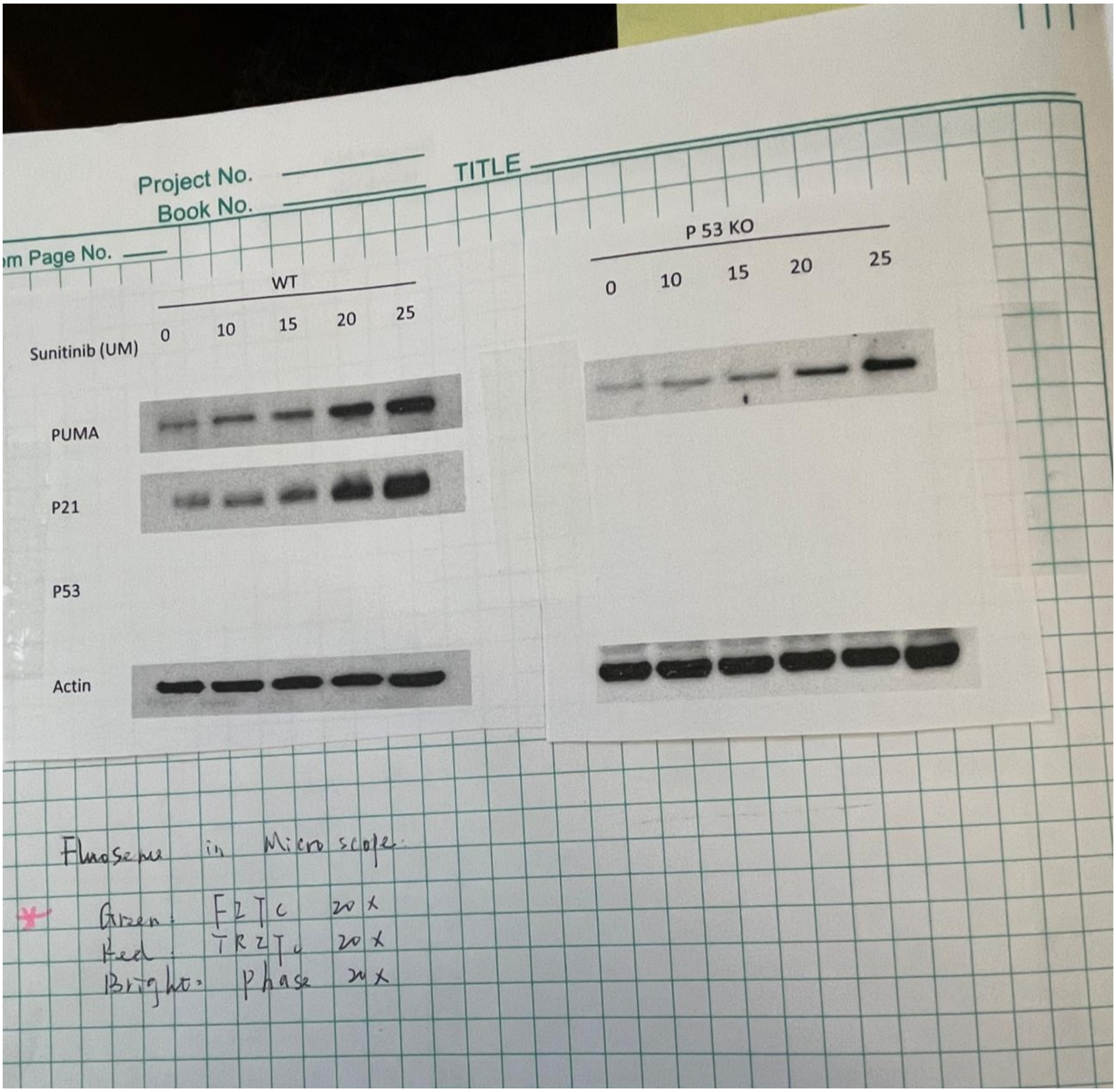

Fig. 1B, film scans

- WT vs p53KO

Actin

p21

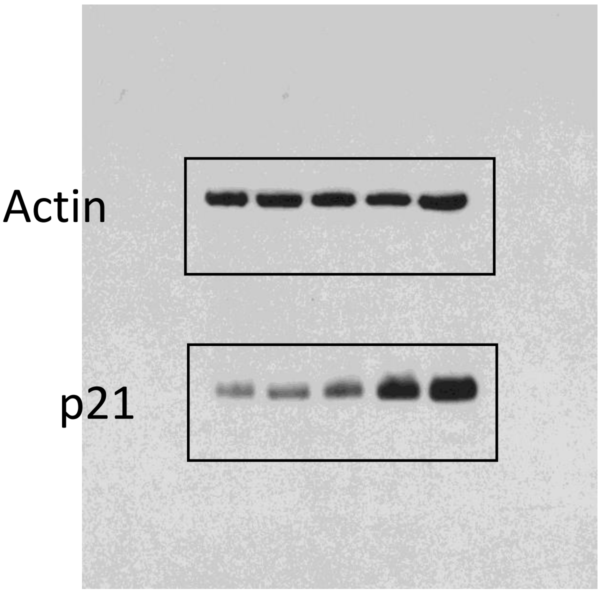

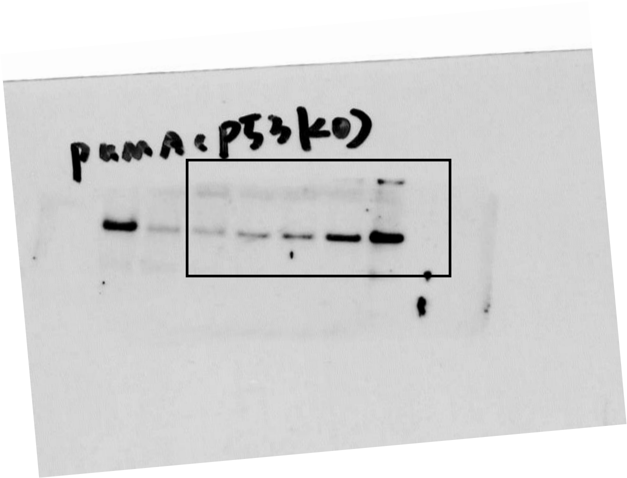

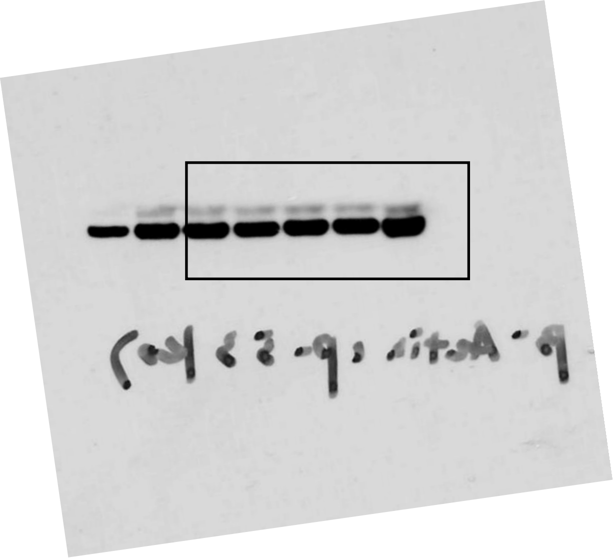

Fig 1C , notebook scan (2/2009)

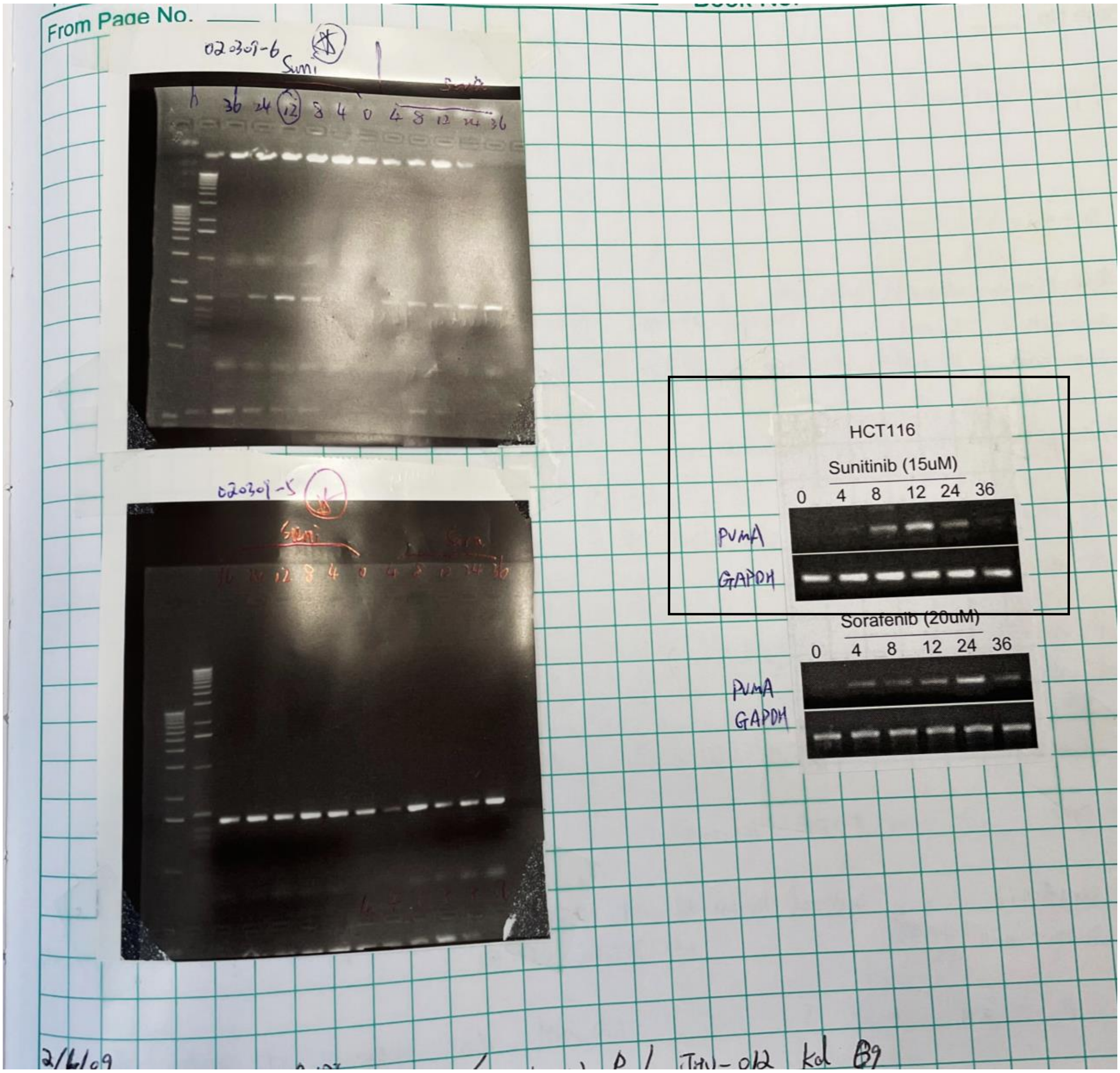

Fig. 1D, film scan

PUMA

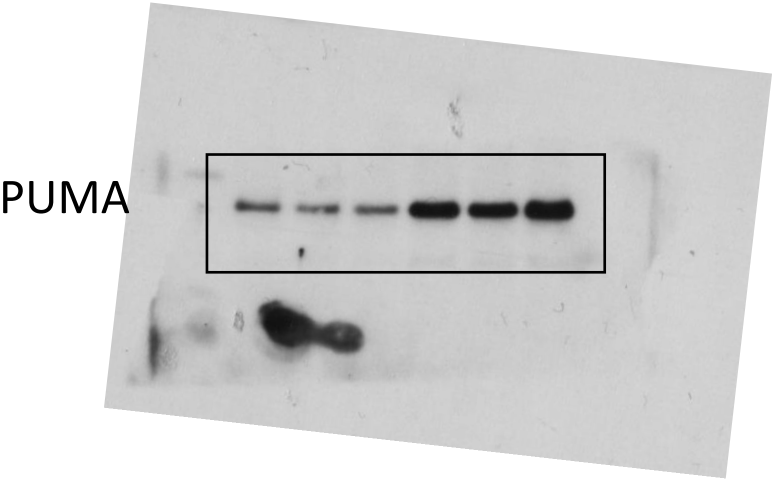

Actin

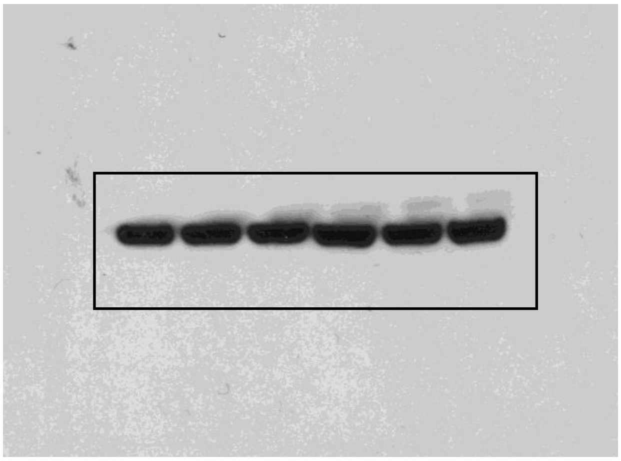

S1A related to 1D.

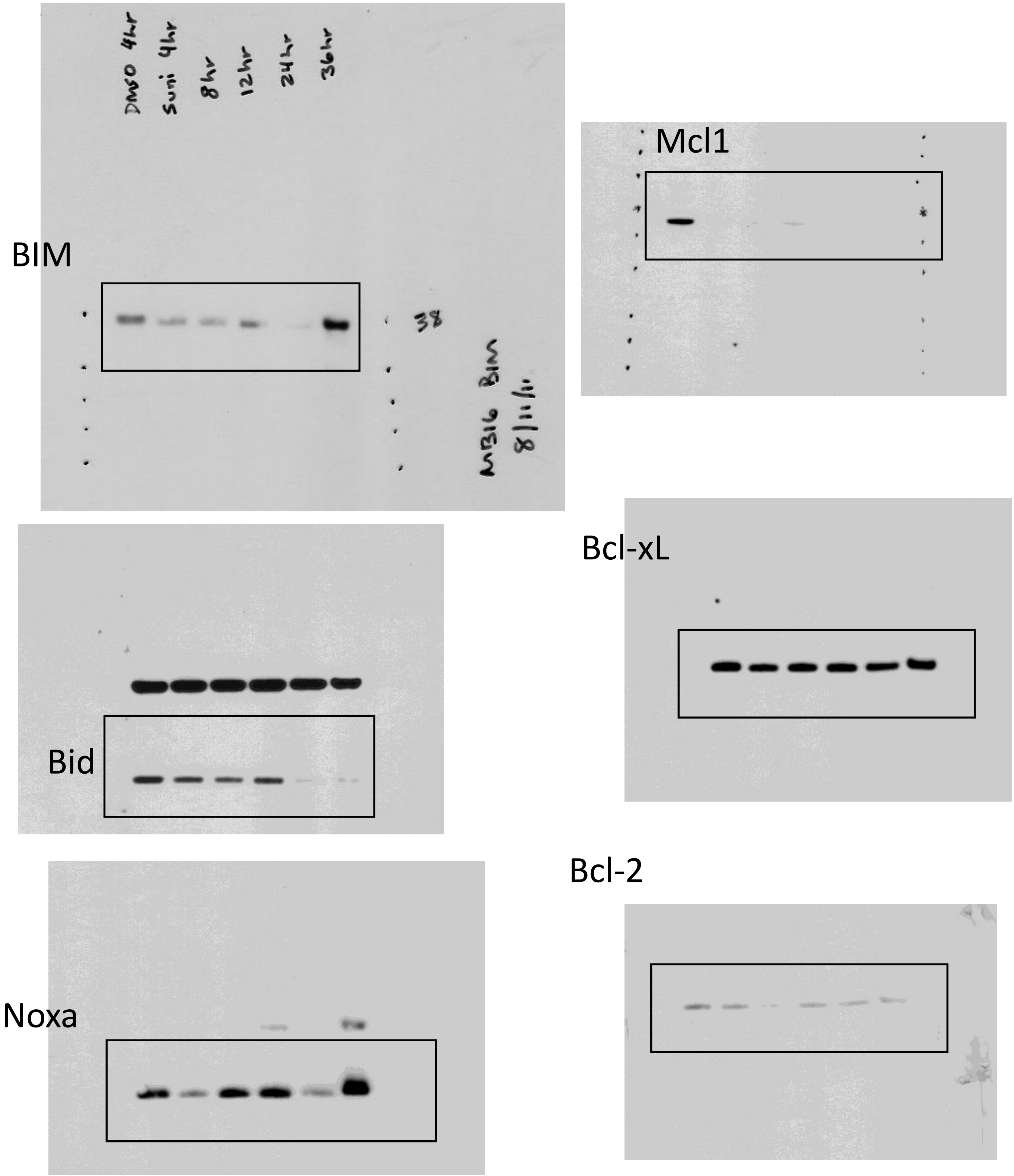

Fig. 2C

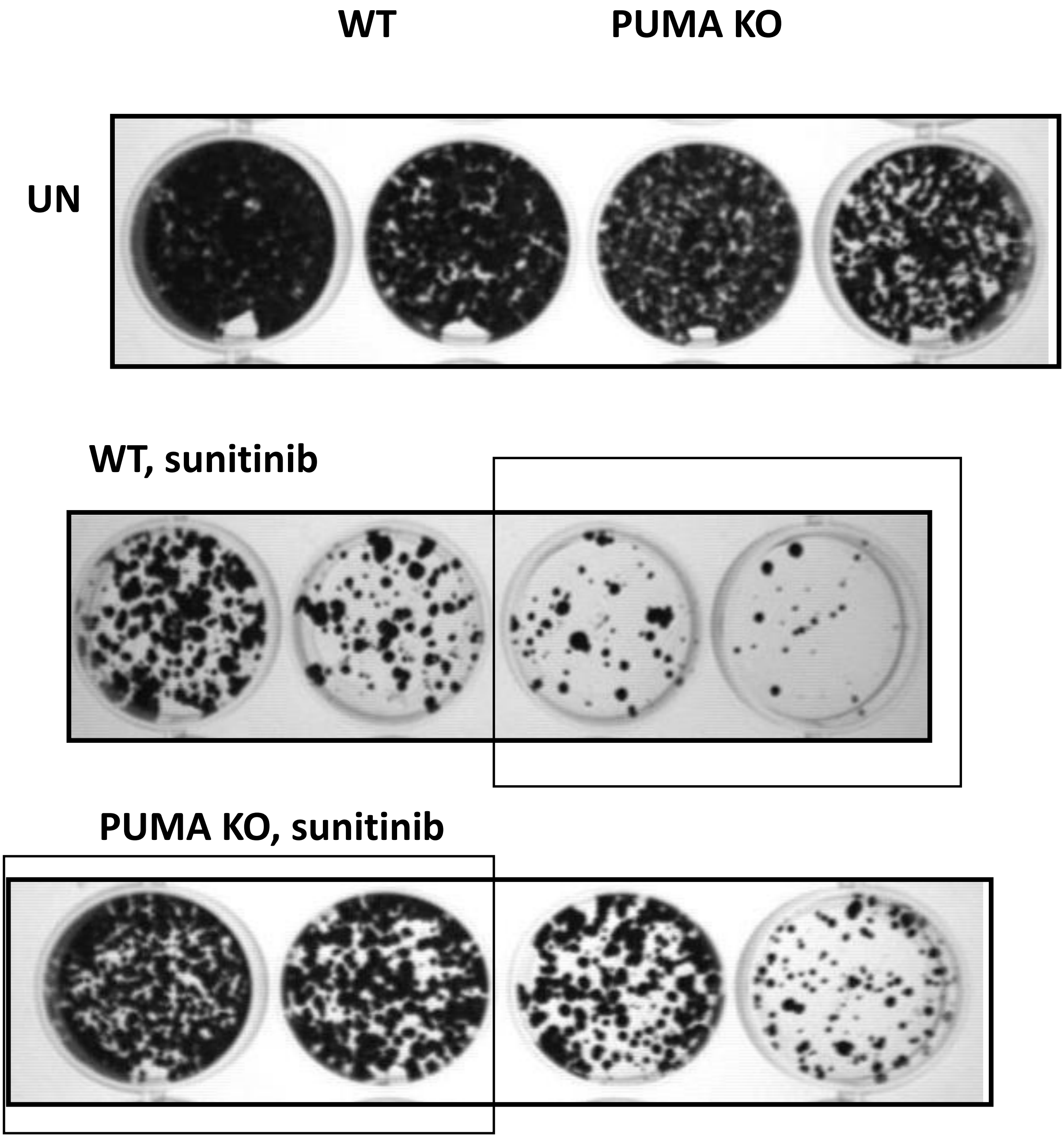

Fig 2D, film scan

PUMA

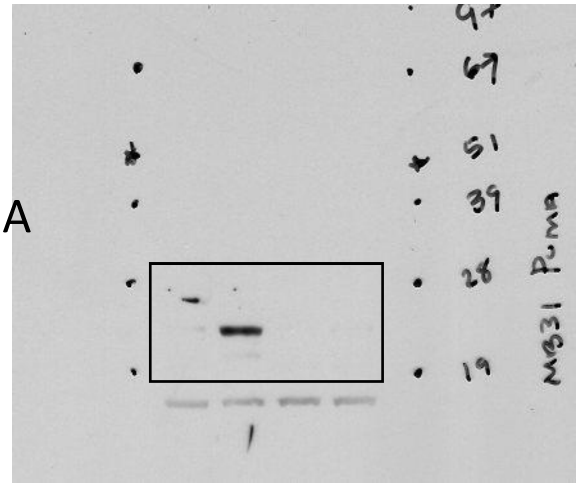

Cas-3

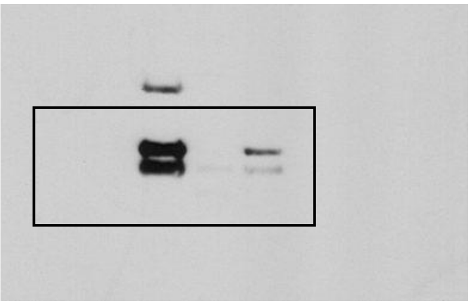

Actin

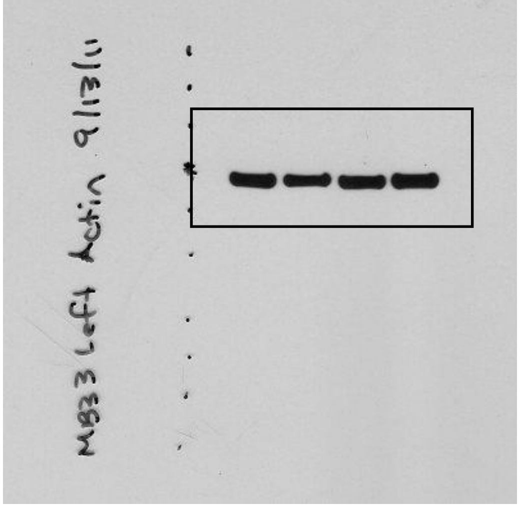

Fig 4A, notebook scan

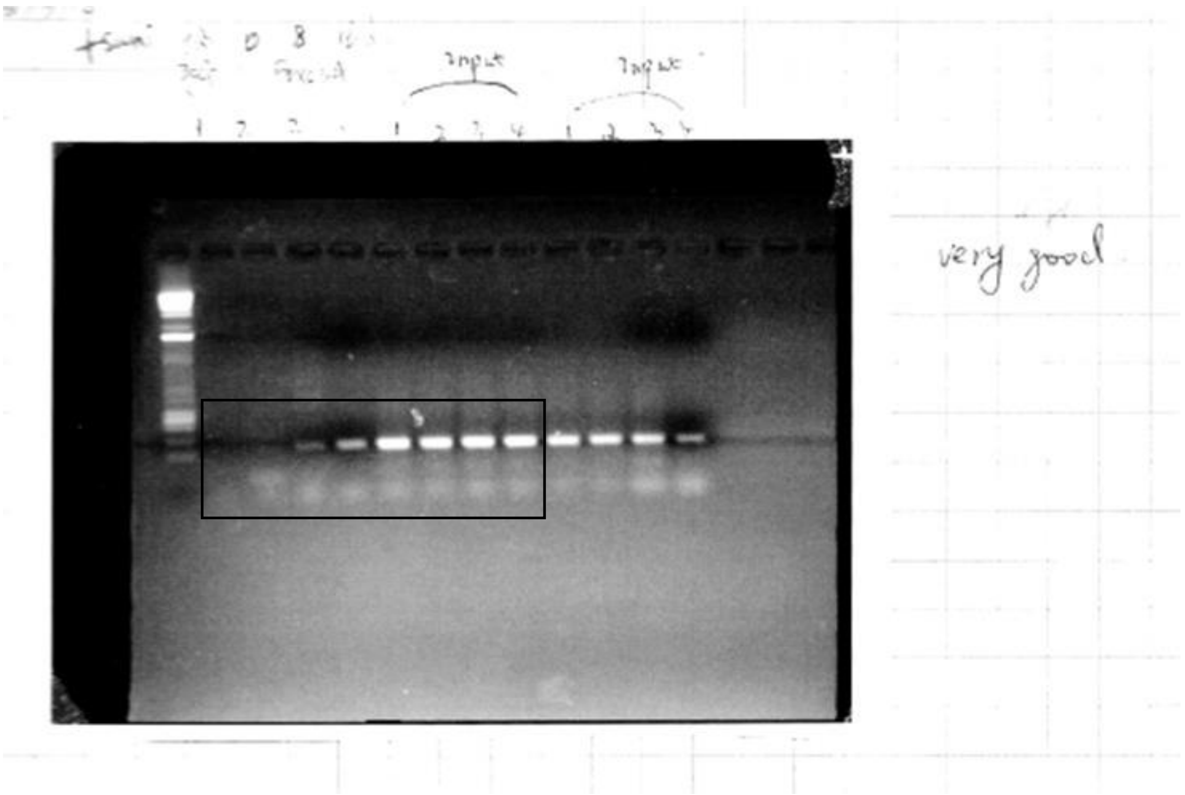

Fig 5 B and C

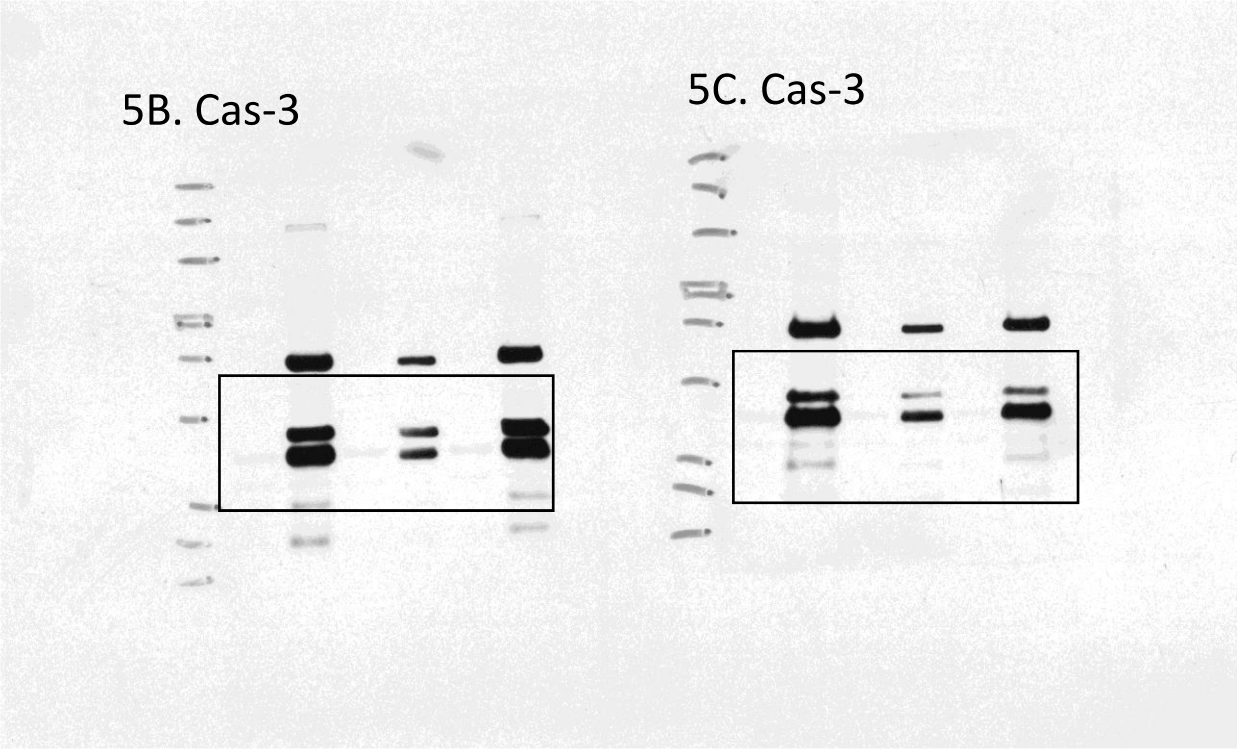

Fig 6B

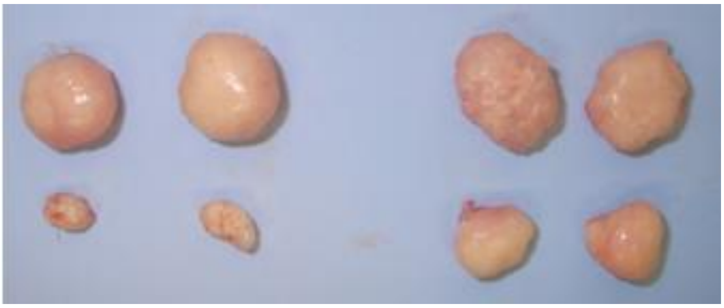

Fig 6C, can not match the zoom area, extra images are provided

pAKT

Con

Suni

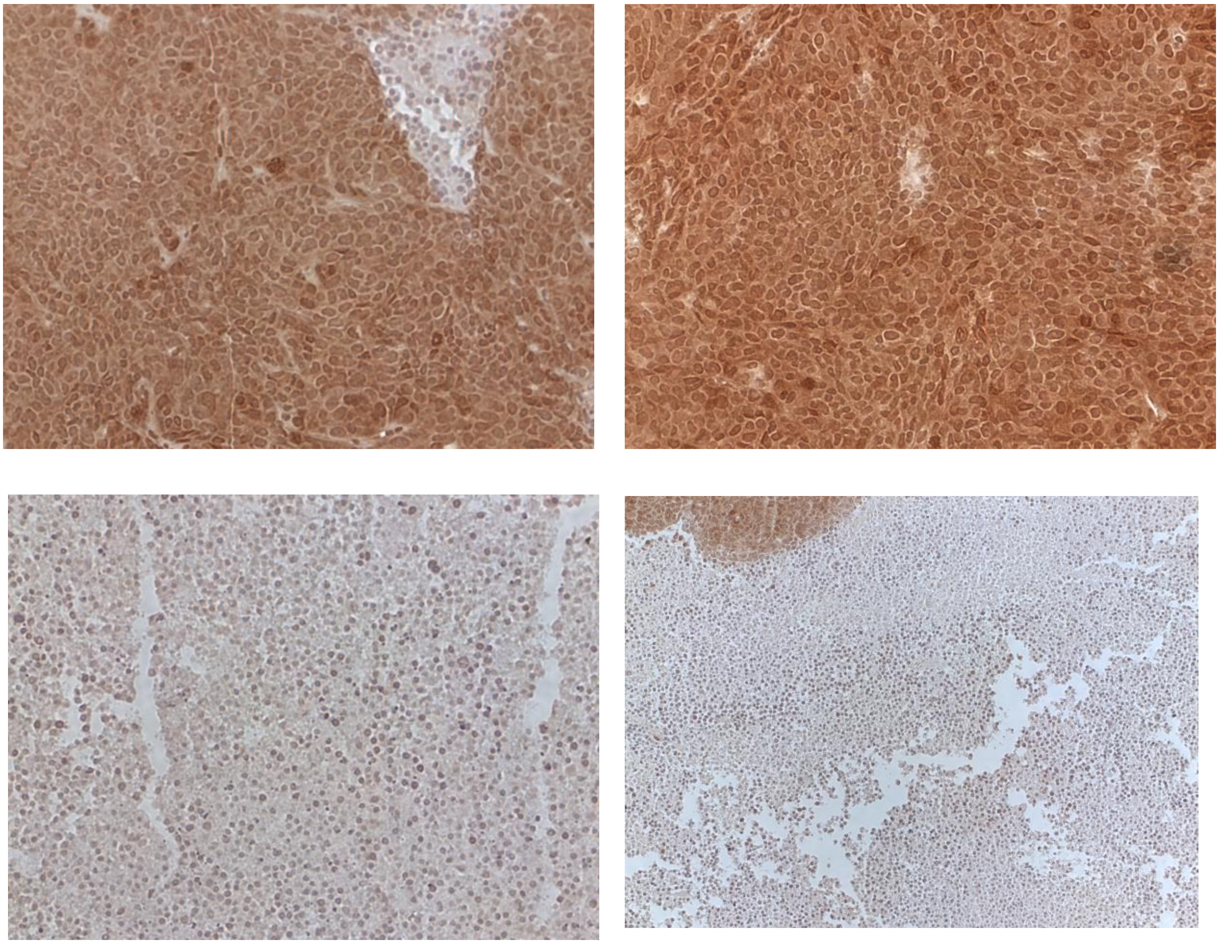

Fig. 6C

Control Sunitinib

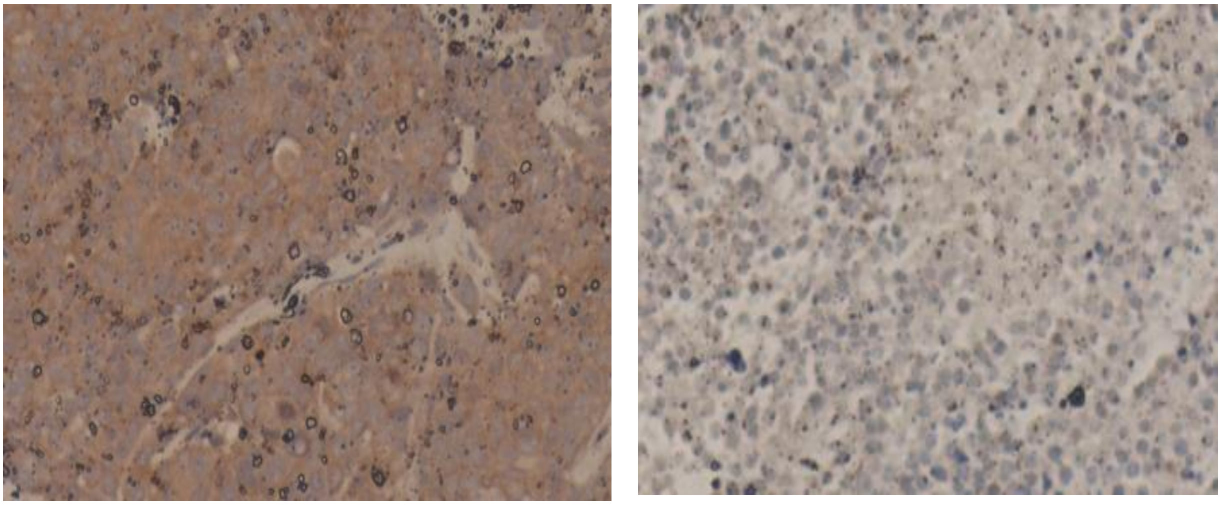

P-FoxO3a
