# Supplementary material for: Editorial Note: The multi-targeted kinase inhibitor sunitinib induces apoptosis in colon cancer cells via PUMA
Source: PLoS One. 2026 Jan 6;21(1):e0339805. doi: 10.1371/journal.pone.0339805 (PMC12773795; doi:10.1371/journal.pone.0339805)
Supplement: S7 File — (PPTX) [file pone.0339805.s007.pptx]

## Slide 1
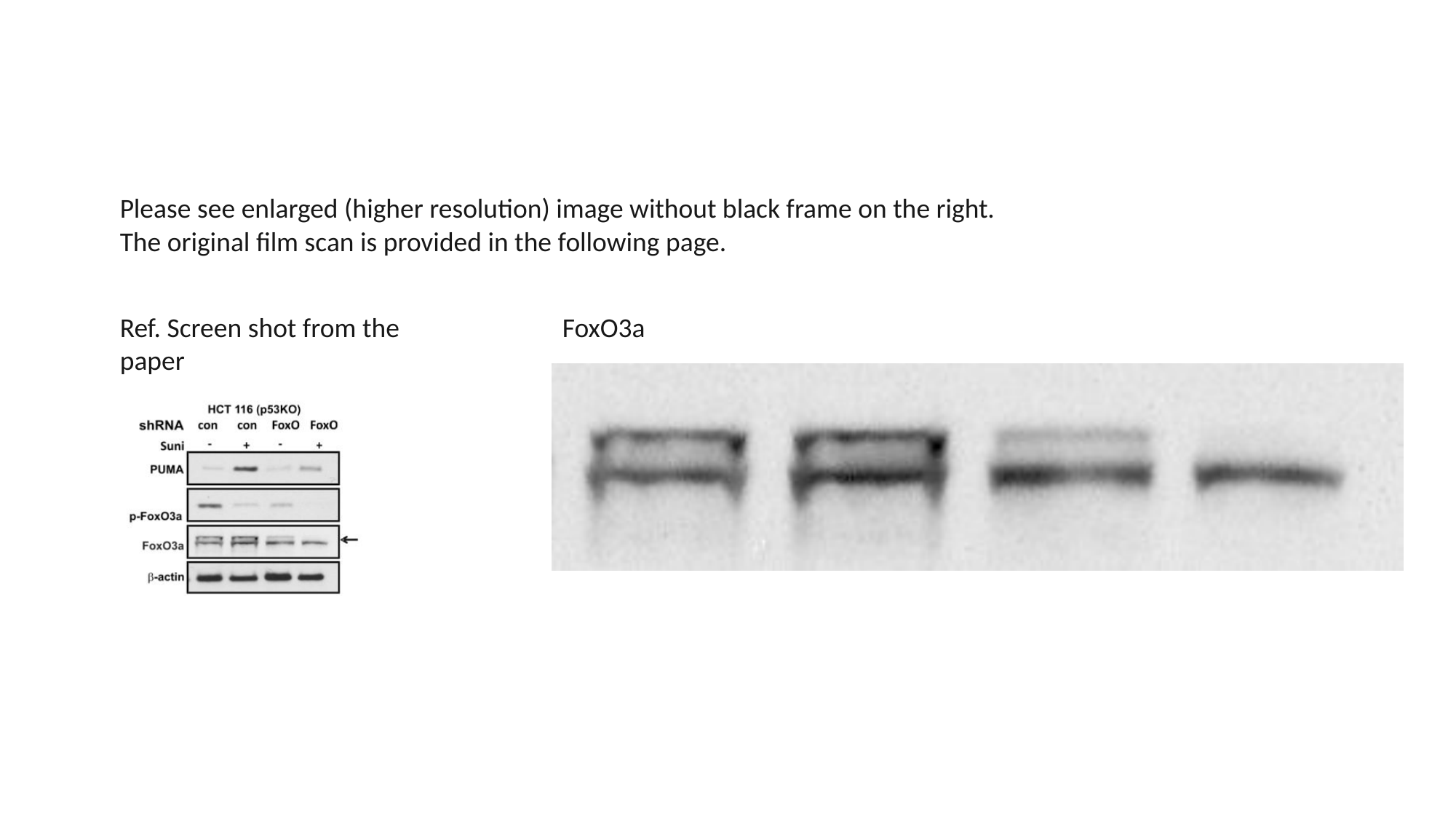

#
Please see enlarged (higher resolution) image without black frame on the right.
The original film scan is provided in the following page.
Ref. Screen shot from the paper
FoxO3a

## Slide 2
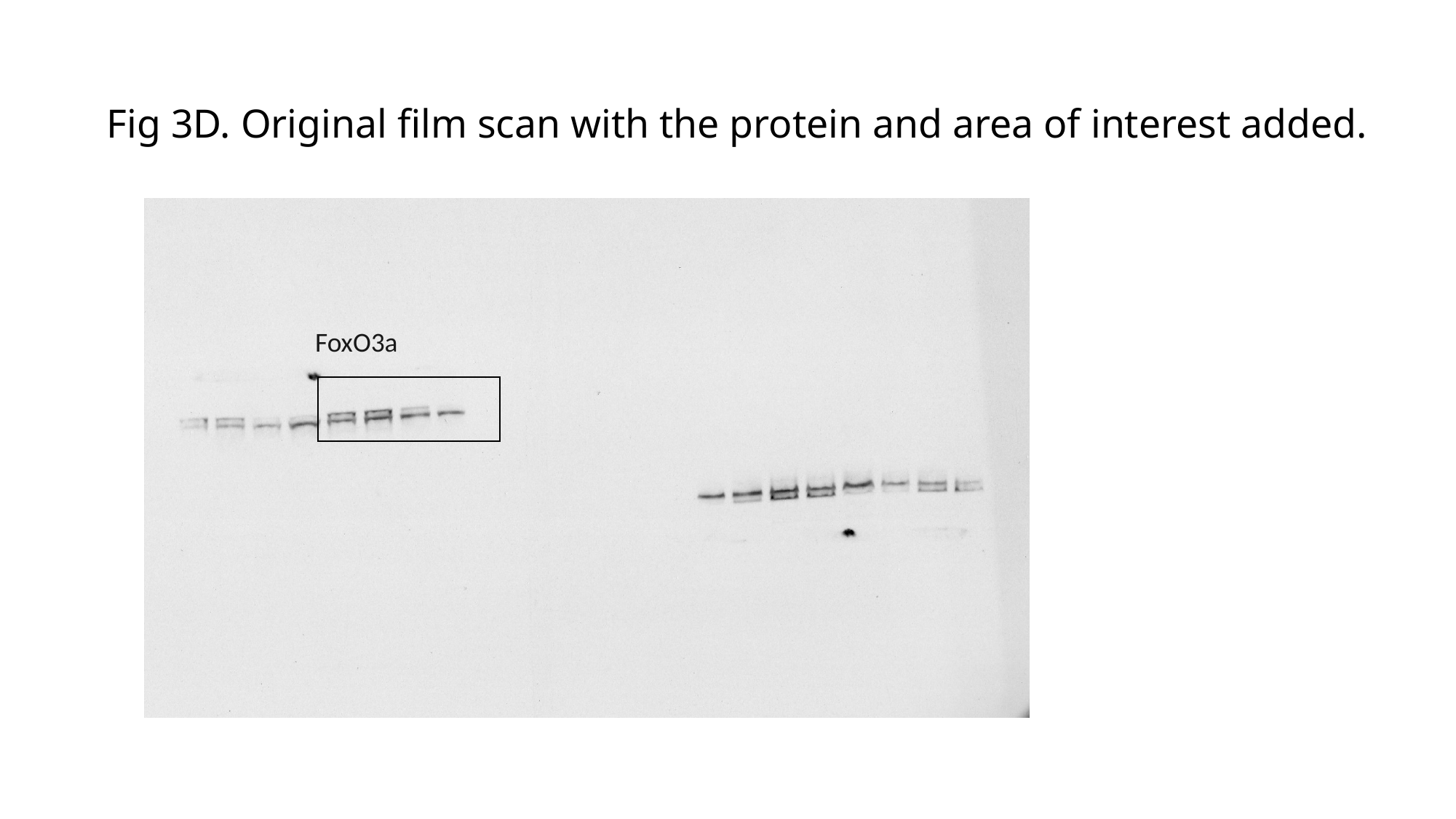

# Fig 3D. Original film scan with the protein and area of interest added.
FoxO3a

## Slide 3
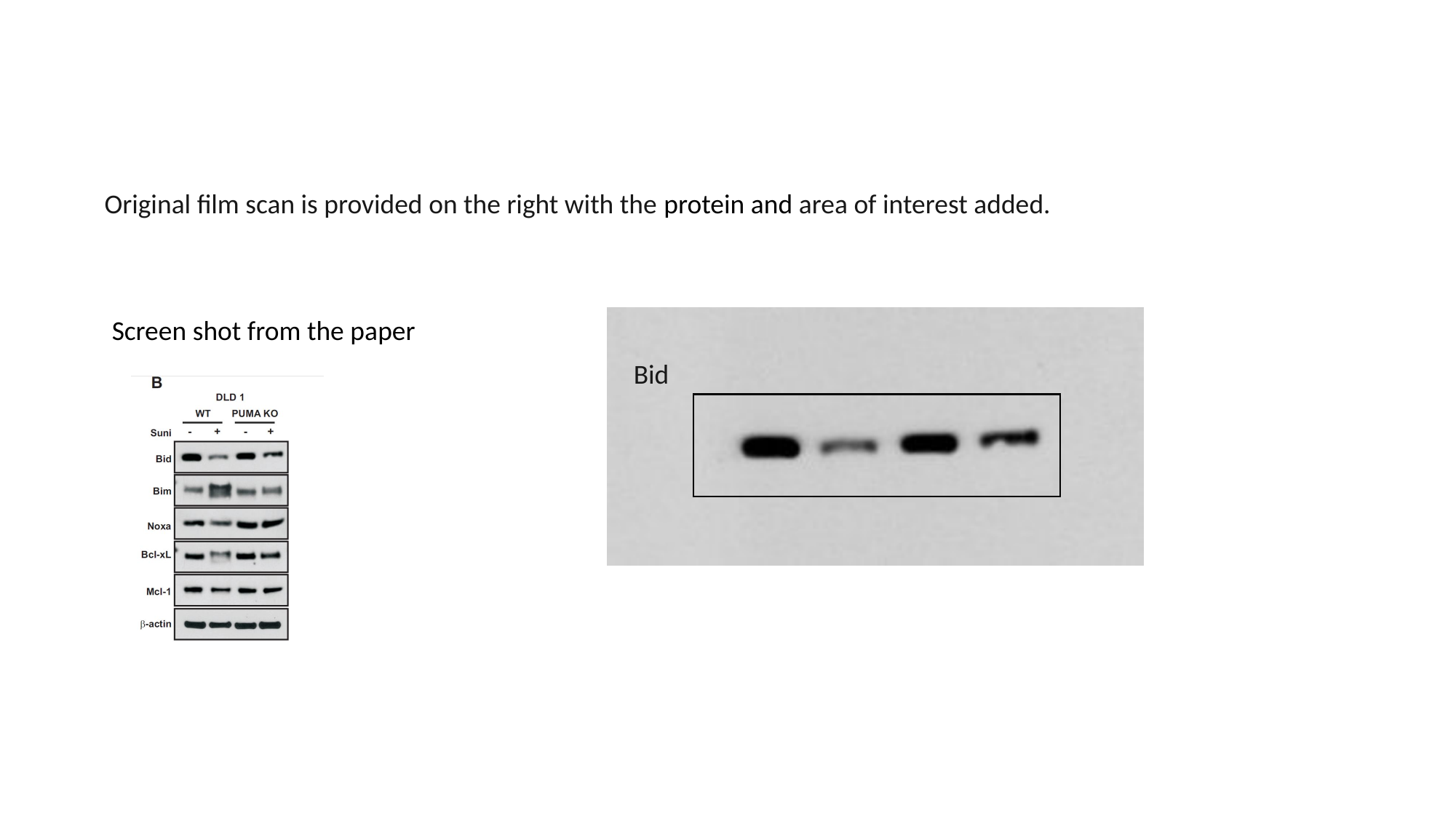

#
Original film scan is provided on the right with the protein and area of interest added.
Screen shot from the paper
Bid

## Slide 4
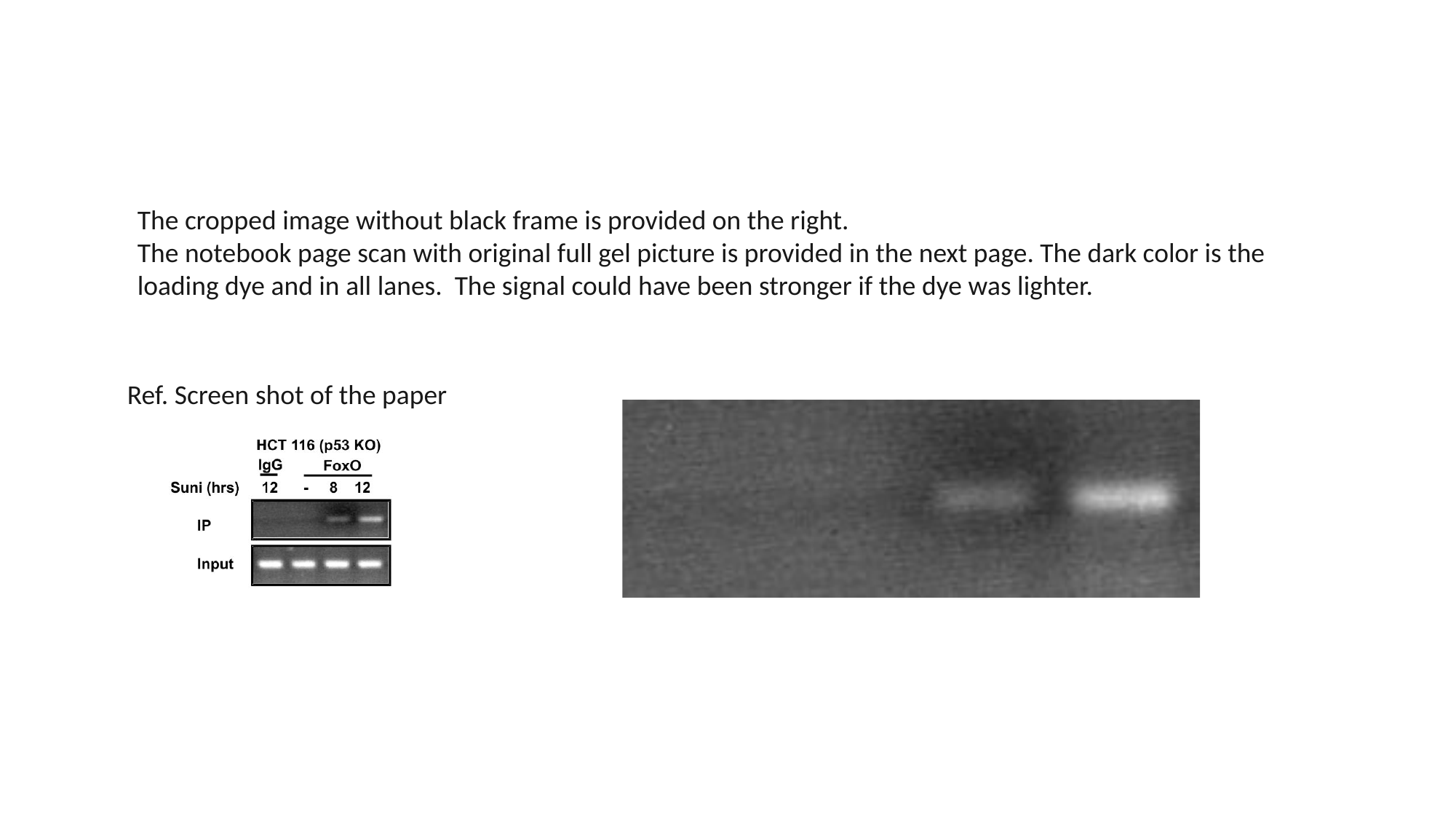

The cropped image without black frame is provided on the right.
The notebook page scan with original full gel picture is provided in the next page. The dark color is the loading dye and in all lanes. The signal could have been stronger if the dye was lighter.
Ref. Screen shot of the paper

## Slide 5
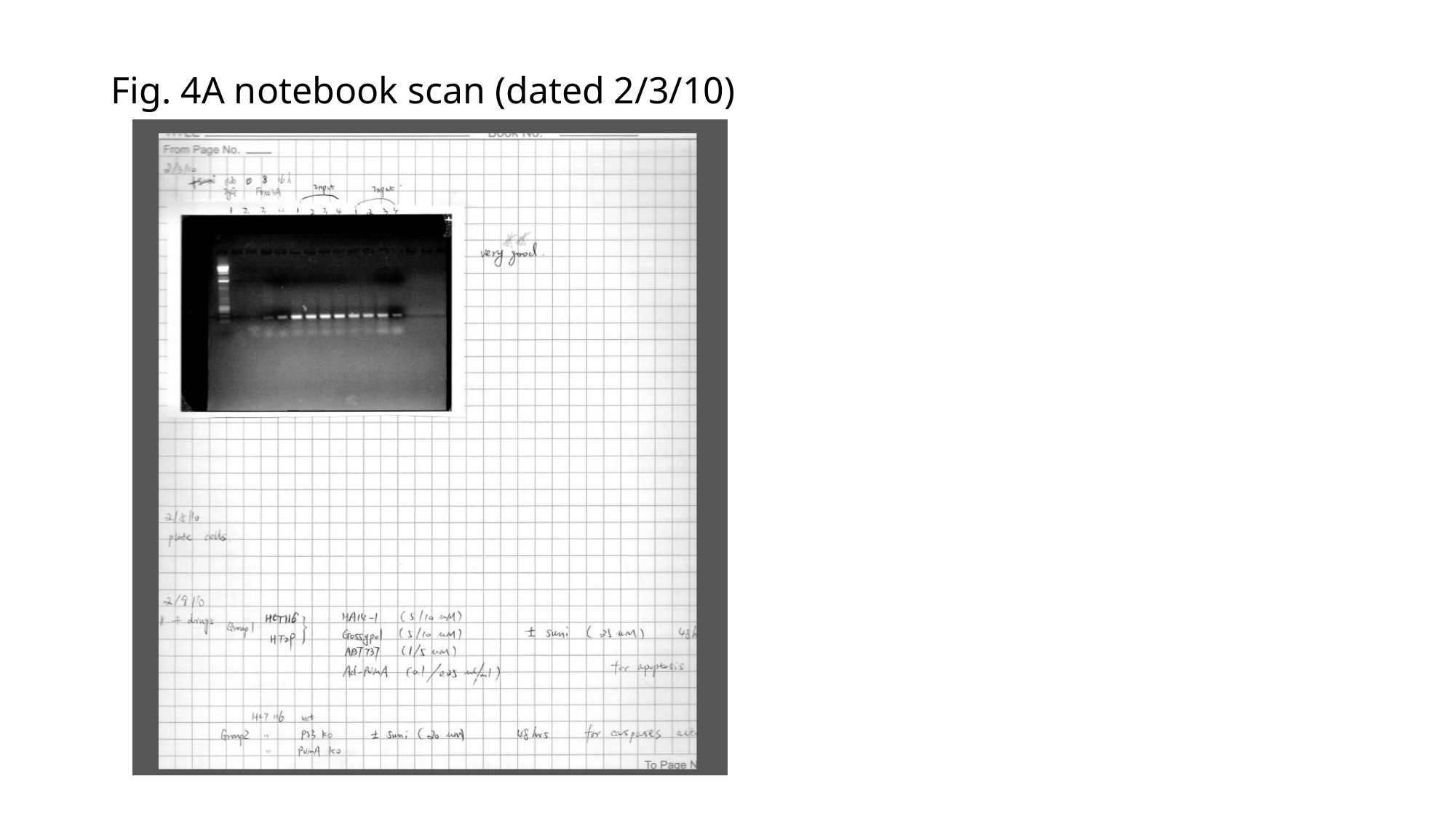

# Fig. 4A notebook scan (dated 2/3/10)
