# Supplementary material for: Editorial Note: The multi-targeted kinase inhibitor sunitinib induces apoptosis in colon cancer cells via PUMA
Source: PLoS One. 2026 Jan 6;21(1):e0339805. doi: 10.1371/journal.pone.0339805 (PMC12773795; doi:10.1371/journal.pone.0339805)

2025 film scans

Article ID 10.1371/journal.pone.004318

S1B related to Fig. 2D. Note same Actin blot as in Fig 2D.

Bid

BIM

NOXA

BCL-XL

Mcl 1

Actin

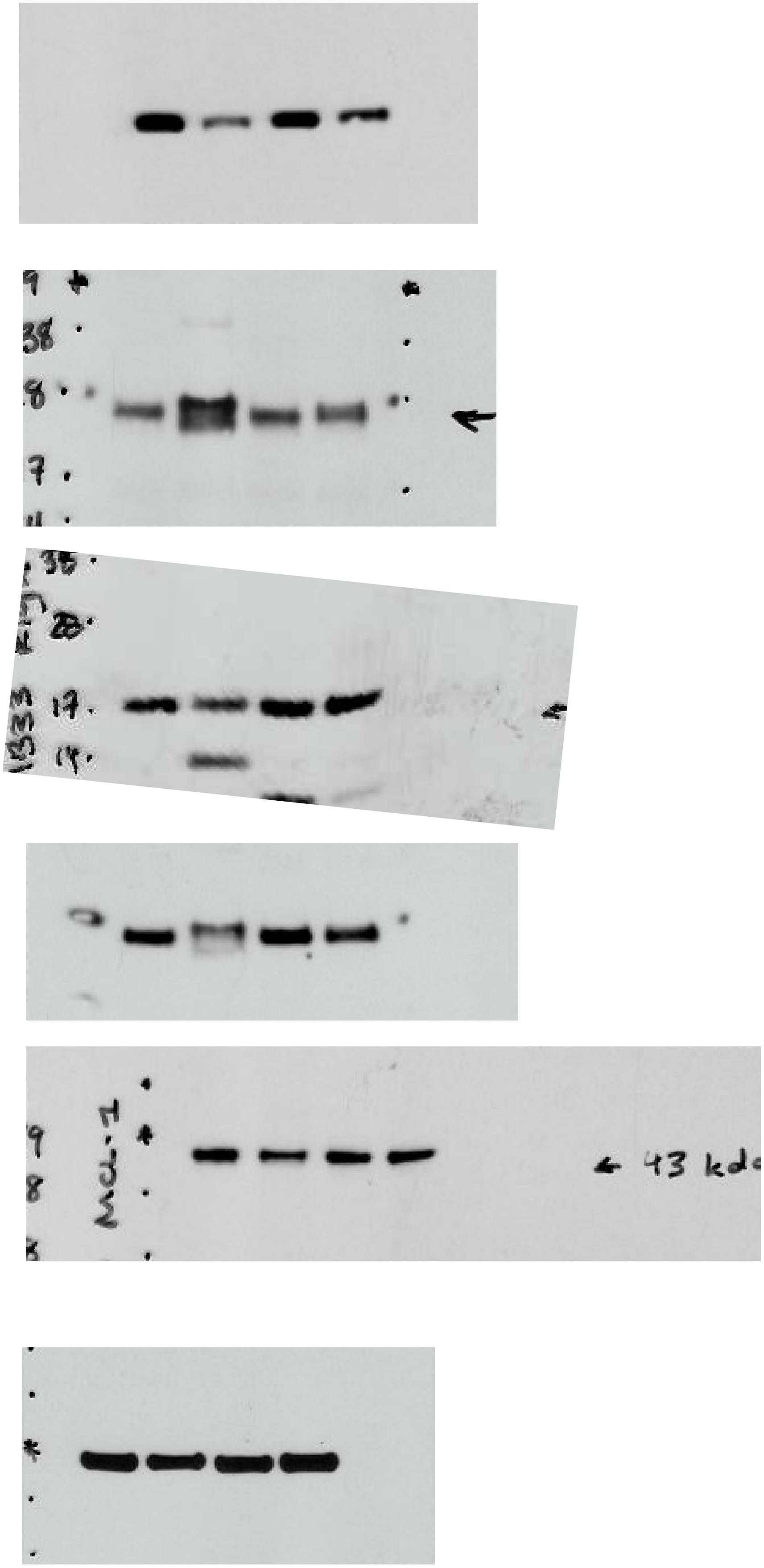

Supplement: S8 File — (PDF) [file pone.0339805.s008.pdf]
